# Hypogene enrichment in Miduk porphyry copper ore deposit, Iran

**DOI:** 10.1038/s41598-022-23501-5

**Published:** 2022-11-09

**Authors:** L. Yousefi Soorani, B. Shafiei Bafti, S. M. Homam, Z. Abbasloo, H. Taghizadeh Zanooghi

**Affiliations:** 1 Geology Department, Ferdowsi University of Mashhad, Mashhad, Iran; 2 Geology Department, Shahid Bahonar University of Kerman, Kerman, Iran; 3 Geology Department, National Iranian Copper Industries Company, Shahr-e-Babak Copper Complex, Shahr-e-Babak, Iran; 4 Exploration Department, National Iranian Copper Industries Company, Sarcheshmeh Copper Complex, Rafsanjan, Iran

**Keywords:** Solid Earth sciences, Geochemistry, Geology, Mineralogy

## Abstract

This study was planned with the aim of identifying the nature and circumstances of the high-graded central core and increasing trend of copper content through depth of 1000 m in Miduk PCD. Mechanisms of high-grading, refer to hypogene enrichment (HE), in PCDs poorly understood. Two main hypotheses for hypogene enrichment formation assumed addition of extra copper to the system, alternatively hypogene leaching and enrichment. In order to obtain alteration-mineralization-geochemical pattern both horizontally and vertically, all macroscopic data extracted from relogging of 6800 m’ drill core along an east–west profile, compiled with microscopic observations from studying of 550 thin-polished sections and copper grades of 3400 samples analyzed by XRF and ICP-OES. Our findings proved hypogene enrichment events at deposit. HE evidences in macroscopic and microscopic scales identified almost as various replacement textures between Fe-Cu sulphides and also vein-reopening by later Cu-mineralization and new generation of disseminated or vein type mineralization. In addition, appearance of dark halo, as consuming intermediate chalcocite phase, around pyrite and chalcopyrite which gradually evolves as bornite, also extruding extra iron as fibrous hematite at the outer edge of bornite product replaced chalcopyrite, partially replacement of bornite and chalcopyrite to hypogene chalcocite and covellite-digenite in deep potassic are other HE evidences in the case study. Here, we draw on microscopic observations and SEM-BSE-EDS results, secondary hypogene genesis for some of bornite and chalcopyrite as a hypogene enrichment evidence. Observations from relogging show that potassic alteration has a relatively good preservation in the center of the deposit from depth to surface, but affected by intense overprinting of subsequent alterations towards margins. Evident function of ore-leaching at margins, also elevated copper grades in central parts of the deposit strongly suggest leaching-fixation mechanism. Where buffer potential of the rock is preserved copper fixation and where it totally eliminated almost complete leaching of copper happened. Consequently, we introduce leaching-fixation as index processes in hypogene enrichment at the case study. We suggest that identifying the nature of hypogene enrichment processes and its characterizations not only improve understanding about PCD’s hydrothermal evolution, but also achieve exploration indicators, furthermore, industrial benefits in the production line.

## Introduction

Porphyry copper deposits (PCDs) are known as low grade-high volume copper resources that supply about 75% world copper^1^ in the mining industry. The deposits typically formed by magmatic-hydrothermal systems intrude the crust as clusters^1^ focused along destructive plate boundaries above subduction zones. In the more highly telescoped systems^1^, successive intrusion of magma as stocks and dykes in one place leads to repeated releasing thermal-hydrothermal fluid cycles, potentially with great chance to generate high-grade porphyry Cu mineralization. High grade mineralization zones in porphyry copper systems and their formation during evolutionary course of deposit have long been considered. High-grade sulphide mineralization in porphyry copper deposits (PCDs) well known such as El Salvador^2^, Escondida^3^, Chuquicamata and Calhausi^4^, Batuhiju^5^, Butte^4,6^, Wafi Golpo^1^. Two main HE generating mechanisms considered as hypogene leaching and subsequent reprecipitation and enrichment of originally chalcopyrite-bearing low-grade protore (0.2–0.5%)^1,6–9^, alternatively addition extra copper to the porphyry system^3^ by hydrothermal Cu-bearing fluid released from later intrusive pulses. The overprinting style of the hydrothermal evolution was noticed as an important factor in the formation of the copper-rich hypogene ore^3^. Overprinting former potassic alteration would happen during declining fluid while a single early porphyry heat source decays^6^, or by fluid recycling due to a later thermal source. In porphyry Cu systems multiphase porphyry intrusions as stocks and dykes are common. Emplacement of each generation of porphyry stocks or dikes could drive a new cycle of alteration-mineralization^10,11^, by discrete fluid release event^12,13^ or at least supply a thermal source^14^. Usually high permeability near the source of the heat responsible for the fluid motion^6^ and re-mineralization. These stocks or dike swarms merge downward into the same underlying pluton^12^ by inward interconnected faulting systems, typically in flower-like structure^15^. Here, there is Important role of structures in deep protore oxidizing-leaching and also movement of copper rich fluid leads to reconstruction genetic pattern of the deposit, specifically very deep structures controlling stock uplift and exhumation and their branches together could work as high permeable deep pathways for fluid motion facilitate more fluid-rock interaction and fluid circulation through deposit controlling protore leaching, copper extraction, remobilization and remineralization processes. Thus, the most central part encounters the most thermal-fluid currents expected regarding the inward faulting system.

Central Iranian Cenozoic Magmatic belt (CICMB)^16^, is one of the major copper-producing regions in the Alps– Himalaya orogeny, including more than 50 copper porphyry-vein type deposits^17^. Most of these resources were formed by subduction and eventual closure of the Neotethys ocean in the late Mesozoic and Cenozoic, synonymous with subduction of the Arabian Plate beneath Central Iran^18–20^ and also form during collisional and other postsubduction tectonic processes^21,22^. In the Urumieh-Dokhtar magmatic arc (UDMA) of Central Iran, magmatism changed from calc-alkaline in the Eocene to adakite-like compositions in the Oligo-Miocene^21,23–25^. Kerman and Arasbaran segments in UDMA and Lut block (east of Iran) are the three most important zones for porphyry and epithermal mineralization. These three zones have great potential mostly for Cu^21,26^, Cu-Mo-Au^26–29^ and Cu-Au^21,26,30,31^, respectively. The first significant porphyry deposits formed in the Eocene and Oligocene^21^ (Lut block^24,26^). However, the main period of porphyry formation (including several large porphyry Cu as Sungun^32,33^ in Arasbaran zone, Miduk^17^, and Sarcheshmeh^20^ in Kerman zone, as well as the porphyry related Sari Gunay^34^ epithermal Au deposit) occurred later in the early to mid-Miocene (~ 20–11 Ma)^21^, synchronous with terminal collision between the Afro-Arabian and Eurasian plates. In fact, most of Iran’s major porphyry and epithermal deposits fall into postsubduction category^21^. Numerous porphyry prospects in the Kerman segment and the Lut block are only partially exposed. Supergene enrichment in most Iranian porphyry deposits is minor, and their relatively low grades reflect hypogene (sulfide) ores, with only superficial oxidation and chalcocite enrichment^21^. The case study, Miduk, is the third most important PCD of Iran located in the Kerman segment as the most prosperous zone for PCD generation (see Supplementary for geological maps and more details; Fig. S1).

Access to an exceptionally One-kilometer exploratory drill core network at Miduk allowed us to investigate more on a high-graded PCD. Here, we compile one kilometer vertical geochemical data and alteration-mineralization pattern in order to interpret central high-graded ore which is continuously increasing through depth. Our observations from relogging show that, in the center of the deposit potassic alteration has a relatively good preservation from depth to surface, but affected by intense overprinting of subsequent alterations towards margins of the pit to the east and west. To altitude of 1500 m asl., at the end of exploratory boreholes with a length of 1000 m, towards center high hypogene copper grades (mostly more than 1%, up to 2.4%) obtained in diorite porphyry protore affected by various degrees of sericite ± chlorite alteration overprinting former potassic that based on this research attributed to hypogene enrichment event in extent of the deposit. Dense fault networks at deposit represent a possible focused pathway for fluid motion, as a consequence high fluid-rock interaction, leaching and remobilization. Although, Individual factors can be effective in HE event, evident function of ore-leaching at margins, also elevated copper grades in central parts of the deposit strongly suggest leaching-fixation mechanism. Regarding this fact that HE-evidences observed vertically at least along one kilometer from the deepest to the surficial levels, presence of late intrusive extended up to near surface to affect upper parts of the deposit seems to be essential, as a possible thermal source for fluid recycling within the deposit.

## Results and discussion

### HE in macroscopic and microscopic scales

**Figure 1:**
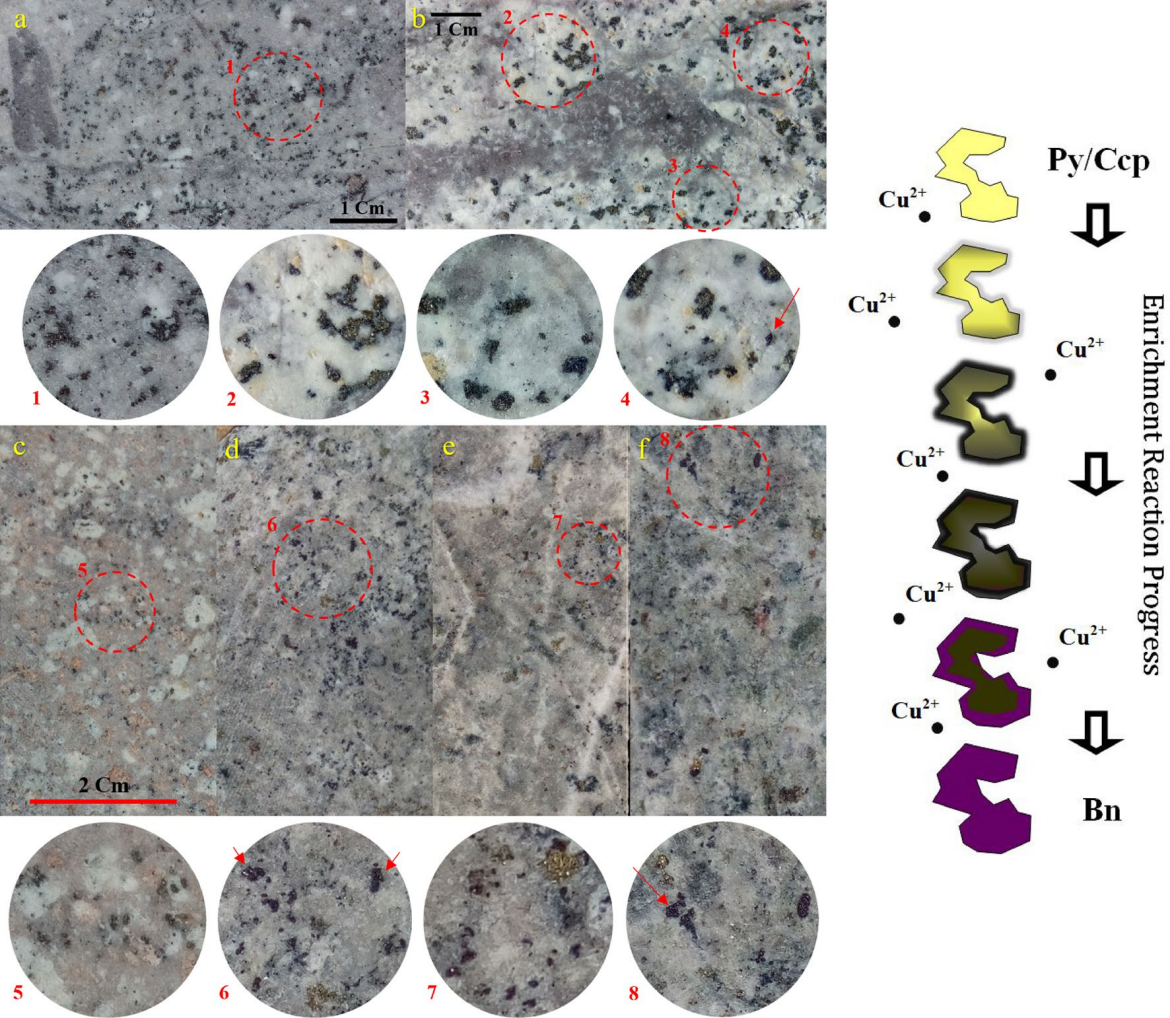
Photographs of fine disseminated pyrite (Py)/chalcopyrite (Ccp) grains replacing to bornite (Bn). Arrows pointing to mature bornite with purple metallic luster. Upper images emphasis on transforming changes started with darken rim of sulfide grains as earthy dark gray halo which in progress of reaction turn to metallic luster, obviously shown in c-f. Aspects of this process are easily recognizable in a brighter matrix of phyllic alteration. sample depth from collar, in meter: (**a**) (777), (**b**) (419), (**c**) (121), (**d**) (888), (**e**) (919) and (**f**) (987). Theoretical equation for replacing reaction of pyrite to bornite with consuming intermediate chalcocite phase can be written as two followed equations; Equation 1: (2FeS_2_ + 5Cu_2_S + OH- = 2Cu_5_FeS_4_ + HS- + 0.5O_2(g)_), Equation 2: (2FeS_2_ + 5Cu_2_S + H_2_O = 2Cu_5_FeS_4_ + H_2_S + 0.5O_2(g)_). Earthy dark luster halo at the outer surface of the parent phase is considered as a first-immature step of hypogene enrichment, that by distance of several meters usually is promising appearance of bornite. In some cases, dark halo and bornite product coexist for individual grains in the same sample. Enrichment reaction progress depicted step by step in schematic model at right-hand, more detailed description provided in the text.

HE mineralogical and textural evidences can be describe as appearance dark halo around pyrite and chalcopyrite which gradually evolve as bornite (Fig. 1), fine disseminated pyrite/chalcopyrite grains rimmed or coated by bornite ± other copper-sulphides, vein-reopening by later copper-rich mineralization (esp. chalcopyrite ± bornite-forming fluid injection to barren A-type quartz veinlets because of their high permeability^1,35–37^), new generation of disseminated or vein type mineralization typically in close association with sericite ± chlorite as overprinting alteration or halo of vein, polymetallic high sulfidation vein, replacement textures between Fe-Cu sulphides as disseminated or vein type, silicate phenocrysts replaced to sericite-copper sulphides (Supplementary, Figs. S2–S10 show evidences in macroscopic and microscopic scales). HE-related veins/veinlets are apparently among latest mineralization phases which cross-cut and/or reopen earlier phases. Our studies confirm that HE event is in close relationship with sericite-forming fluid overprinting. Brimhal^6^ noted that vein sericitic halo is one in which the copper-bearing protore sulphide, chalcopyrite, is progressively leached and more disseminated chalcopyrite destruction more intensive sericitization would occur in K-silicate protore. However, in many cases enrichment as bornite/chalcopyrite formation is in close association of sericite/muscovite in the text of alteration or halo of vein/veinlets. New HE mineralization is also found as fracture-filling irregular sinusoidal to branched hairy veinlet/microveinlets of chalcopyrite/bornite or both with or without sericitic halo around the vein. To deepest drilling in deposit variable assemblage of pyrite, chalcopyrite, bornite, chalcocite, digenite, covellite, molybdenite, rarely sphalerite and galena identified in hypogene enrichment event. Presumably relating to texture destructive effect of phyllic as general alteration at moderate levels (~ 400 m) to the surface, there is a considerable reduction of phenomenon in the form of vein-veinlets whereas frequency of disseminated increase in high sericitized to phyllic context mainly including pyrite, bornite and less chalcopyrite, chalcocite, digenite and covellite. This mineral assemblage could be named pyrite-bornite zone as mentioned in Gustafson and Hunt^2^. Experimental study by Kojima and Ueno^38^ predicted temperature range 350-250ºC by using near-neutral hydrothermal solutions as formation condition of the pyrite + bornite assemblages. At upper levels close to the surface, supergene enrichment gradually is added to the complex. Here, Supergene zone considered the levels with presence of sooty chalcocite coating on thick pyritic vein, chalcantite and more common turquoise as disseminated or vein type. In studied profile supergene enriched zone thickness varied from zero in central high graded ore to a maximum vertical extent of 130 m at low grade eastern margin of the pit, regarding to ubiquity of turquoise, chalcantite and sooty chalcocite, although local presence of sooty chalcocite continued to 280 m from surface at marginal areas. Vertically, HE evidences observed from surficial to deepest levels, and its horizontal frequency varied from low in marginal areas to high in most central parts of the deposit. In practice, as mentioned before by Ossandon et al.^4^, fine-grained Cu-coated disseminated pyrite as HE evidence leads to recognition difficulties in deposits that also underwent supergene Cu sulphide enrichment, therefore hypogene contribution is commonly not distinguished from the supergene enrichment products^1,39^.

Observed hypogene enrichment phenomenon mainly is in the form of replacement of the outer rim of pyrite and less chalcopyrite by bornite, which in some cases led to almost complete replacement. The conversion process in successive meters of logging studies can be tracked and investigated. Replacement begins by appearing an earthy luster dark halo around pyrite or chalcopyrite (Fig. 1). This narrow dark halo is an unstable intermediate phase with dark effect on finger touch. One sample analyzed by XRD indicates related mineralogy as chalcocite and digenite (Supplementary, Fig. S11). Gradually the opacity becomes darker and earthy luster turns metallic with a dark-very dark blue or purple tune or a mixture of both (Fig. 1). Matching data from hand specimens and microscopic studies showed that the purple and dark blue lusters in natural light belong to bornite and chalcocite-covellite-digenite ± bornite, respectively. We strongly suggest this early HE evidence as the first key indicator in high-grade deposit investigations. Here, we predict the theoretical equation for replacing reaction of pyrite to bornite with consuming intermediate chalcocite phase as Eqs. (1–2) (Fig. 1; legend, and Supplementary Table S1). However, previous experimental study^40,41^in consistent with our findings confirms generation of black suspension of chalcocite at first steps of reaction of bornite replacing chalcopyrite which disappeared in higher reaction extents. Furthermore, in another experimental study conducted by Berger et al.^5^, pyrite plus chalcocite used together as consumption material in synthesis bornite.

On a microscopic scale, the hypogene enrichment process is identified as a multiphase event at the deposit. Pyrite with rim of chalcopyrite/bornite as disseminated grains or in veins show common form of enrichment. In veins pyrite is usually brecciated and copper-bearing fluid fills the spaces mostly with chalcopyrite/chalcocite (Supplementary, Figs. S9, S10). Brecciated texture in pyrite probably generated by hydrostatic pressure of late ore-bearing fluids.

Rimmed replacement texture (Supplementary, Figs. S4, S8) is the most common, but in some cases, replacement appears as inclusions of chalcopyrite and/or bornite within pyrite (Supplementary, Figs. S7, S8) or rarely as exsolution-like texture of bornite lamellae within chalcopyrite lattice. Bornite formation along cracks in special directions within chalcopyrite suggests preferential replacement in the twinning plane of chalcopyrite^41,42^, also called herringbone texture^41^ (Supplementary, Fig. S7). Moreover, another form of replacement is observed as exsolution feather-like texture of covellite, chalcocite and chalcopyrite mixture at replaced margins of bornite (Supplementary, Fig. S7). This oxidation of bornite to more copper-rich sulphides associated with extruding chalcopyrite as back replacement^43,44^. In some cases, typically, remnants of chalcopyrite can be seen in reaction front of pyrite to bornite (Supplementary, Fig. S8). Further, replacement of bornite to chalcocite, subsequently to covellite-digenite both in veins and disseminated grains can be interpreted as evidence of progress in the enrichment process (Supplementary, Fig. S9). To the depth chalcopyrite is the most common sulphide, and the core of sulphide replacement (parent phase) changed from pyrite to chalcopyrite towards central deeper levels of deposit, resulting in the highest copper grades.

Because of widespread influence of overprinting, HE evidences can be seen through the entire deposit but in different frequency and intensity. In the sericitic halo of vein/veinlets presence of pyrite, chalcopyrite and bornite and replacement textures is common. Where overprinting alteration is not effective, for example in text of potassic by away from sericitic halo of vein/veinlets, chalcopyrite is the common sulfide mineral with no replacement evidence. At the low-grade eastern margin of the deposit, in hypogene phyllic, pyrite is prevalent as the principle sulfide mineral, chalcopyrite is low and bornite is rare. Most pyrite grains show no replacement. HE evidences almost limited to formation of a thin layer of chalcopyrite and much less bornite as inclusion or rim replacement textures in some of disseminated pyrite. Towards the central part of the deposit, pyrite content decreases and predominant sulfide gradually switches to chalcopyrite. At high-grade central parts, abundant bornite exists as a secondary hypogene product of chalcopyrite and pyrite replacement. In some cases, more copper-rich HE products such as hypogene chalcocite and covellite-digenite partially replaced bornite and chalcopyrite in deep potassic central parts. Bornite replaced to chalcocite-covellite phases commonly from inside of grain.

As is common, replacement reaction starts at the outer surfaces of parent grain and proceeds toward the core. In some cases, the progressed reaction front shows preservation of grain shape (Supplementary, Figs. S7, S8), suggesting sweeping and monotonous replacement rate. Sometimes the intensity of replacement is so much that leaves only minor traces of previous minerals and almost completely covers the parent phase. However, two or three enrichment phases can be detected in each thin section. In many cases, presence of fine molybdenite laths, mainly in the text of the sericitic halo (Supplementary, Fig. S6) and sometimes inside the veins adhering to chalcopyrite and bornite rim over pyritic core, and also limited formation of galena and sphalerite on the edges of the replacement products, Cu-sulphides, (Supplementary, Fig. S10), indicate simultaneous deposition of other elements in addition to copper and reveal polymetallic enrichment process at the deposit. Presence of anhydrite as a major paragenesis in this high-grade mineralization indicates ineffectiveness of meteoric water in transport and loading of metals.

### HE characterization advantages

However, knowledge about vertical and horizontal mineralogical distribution and grade intensity variations attributed HE event in deposit has great importance in several ways. First of all, leached and enriched ores significantly affect the exploitation-feed program because of sharp copper grade variations. Second, flotation recovery and concentrate grade deprive by presence of floated pyritic core coated by copper sulphides which resulted in enrichment event, the most common and problematic form of that in ore dressing known as pyritic-core bornite, which further have negative effects on smelting by extra iron availability (in floated pyrite structure) for magnetite formation. Add to coated/rimmed texture as the most common replacement feature, inclusion texture that connects pyrite to Cu-sulphides (in very fine grinded particles) also capable of increasing active pyrite, a big problem for flotation concentrates and recovery. Albeit, more copper content as pyrite-replaced form in high-graded PCDs, encountering more recovery problems, too. At case study, as a consequence of high overprinting sericite alteration more pyritic core exist at margins of deposit, whereas towards deep central core pyrite abundance decrease, inversely chalcopyrite as most common sulphide elevated in core of replacements, favorable to flotation concentrates. Furthermore, HE indicators for exploration high-graded deposit would depict based on alteration-geochemical pattern as leached-depleted and high mineralized-enriched zones. On the other hand, presence pair of bright high-sericitized porous zones with no obvious mineralization and high bornite mineralized zones should be considered as HE evidence. Other clues are the dark halos around pyrite and chalcopyrite as an early stage of bornite formation in the enrichment process, also microscopic evidences as ore grains with pyritic core, moreover ubiquity of Cu-Fe sulphide replacement textures.

### Copper enrichment in SEM–EDX results

**Figure 2:**
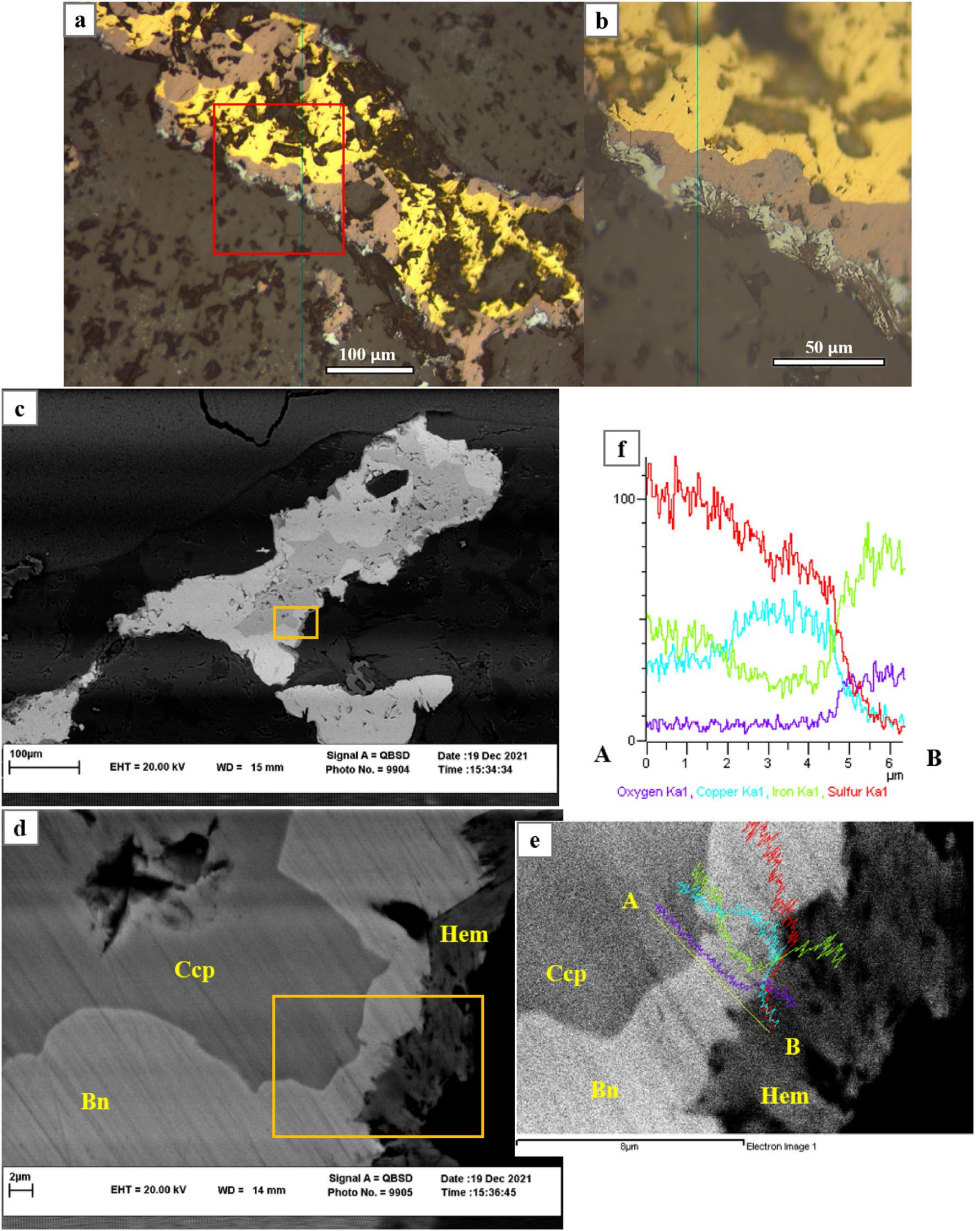
Copper enrichment as replacement of chalcopyrite by bornite in the veinlet and removal of extra iron as hematite (Hem) at rim of reaction. (**a**, **b**) Hematite formed after chalcopyrite replaced to bornite, reflected light. (**c**–**f**) BSE images and EDS analysis micrographs shows smooth and gradual increasing in copper and decreasing in iron and sulphur content in the border passing from chalcopyrite to bornite. Sample; DDH: MDK-39 depth from collar: 891 m. Overall reaction for replacing chalcopyrite by bornite and hematite can be written as two followed equations; Equation 3: (2CuFeS_2_ + 3Cu^2+^  + 0.75O_2_ = Cu_5_FeS_4_ + 0.5Fe_2_O_3_), Equation 4: (5CuFeS_2_ + 6H_2_O = Cu_5_FeS_4_ + 2Fe_2_O_3_ + 6H_2_S).

For more confident and eliminate any doubt about relationship between adjoin phases with replacement texture, detailed scanning electron microscopy with energy dispersive X-ray analysis (SEM–EDX) applied for determining variations of principle components (Cu, Fe, S), along short scan lines, on a few micron scale, vertical to the border of each two phases. However, assuming paragenesis relationships sharp elemental variation along boundaries between two individual phases is expected, whereas for those of replacement phases one to another predicted as gradual. Target grains are chosen among both straight and curved boundaries of pyrite- chalcopyrite, chalcopyrite-bornite and grains covering progressive replacement between all these three phases. For all cases results show that copper is gradually increasing passing through parent phase to the more copper-rich product, confirming replacement as copper enrichment function at deposit. Copper increases accompanied by decreasing sulfur and iron contents in product phase rather than parent phase (Fig. 2, and Supplementary, Figs. S12, S13). In a previous experimental study^41^ on replacing chalcopyrite by bornite, regarding absence of Fe-phase apart from chalcopyrite and bornite, Fe and S conservation as overgrowth of product on the outside of the grain considered as the most likely assumption. However, based on observations in this research we found that transforming chalcopyrite to bornite accompanied by extruding extra iron as fibrous hematite at the outer edge of the bornite product in some veins and disseminated ore grains (Fig. 2). Overall reaction for replacing chalcopyrite by bornite plus hematite can be written as Eqs. (3–4) (Fig. 2; legend). Some previous experimental studies on Fe-Cu sulphide replacement and resultant reactions (Supplementary, Table S1) are in consistency with our mineralogical findings related to a natural hydrothermal system.

### Alteration overprinting and HE pattern in extent of the deposit

Hypogene enrichment extends to the deepest part of the deposit. Gustafson and Hunt^2^ has also reported the roots of the pyrite-bornite complex to the lowest levels within the Kfeldspar-biotite alteration zone. It seems even at the deepest part of potassic alteration replacement events related to sericitic and sericite-chloritic overprinting alterations. However, in some cases, with very poor coverage and limited presence of sericite, replacement and enrichment developed as well.

**Figure 3:**
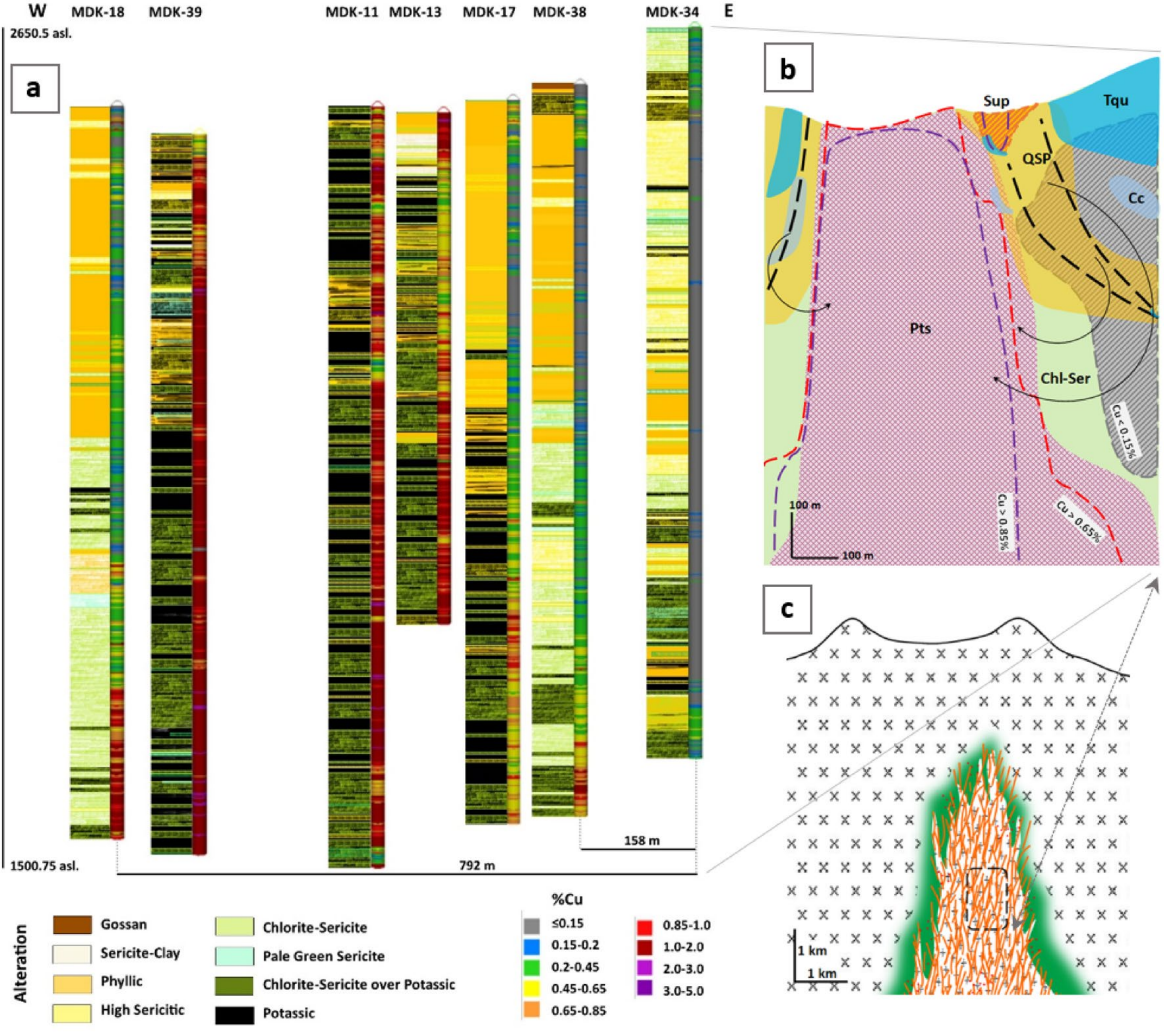
Alteration and copper grade variations along east–west profile (see Supplementary, Fig. S1, profile projected on geological map). (**a**) Depletion of copper toward corners of deposit to the east and west well correlates with intensive overprinting of phyllic over potassic alteration which has to be caused by deep structural fluid infiltration and leaching influence. Supergene enrichment in upper levels of MDK-13 bore hole associated with intense sericite-clay rich alteration. (**b**) Leached-enriched schematic model sketched based on (a), and extra descriptive data. The most depleted areas show in grey. Presence of turquoise and sooty chalcocite confirm effectiveness of local supergenic processes. Conceptual faults and recycling Cu-rich fluid paths depicted by black dashed lines and curved arrows, respectively. (**c**) Approximate location of studied profile on the hypothetical cross section (adopted after Burnham^45^) of intruded Miduk porphyry, mineralized primarily in stockwork potassic and narrow propylitic (green halo) alteration zones. MDK (Miduk), Cc (Chalcocite), Chl-Ser (Chlorite-Sericite), Pts (Potassic), Sup (Supergene), QSP (Quartz-Sericite-Pyrite), Tqu (Turquoise).

Effectiveness of sericitic alteration overprinting on copper enrichment process has been significant, so that, in the central part of the deposit affected by relatively weak sericitic alteration overprinting, where predominance alteration of rock is still potassic, enrichment process occurred with abundant secondary hypogene bornite resulted in high grade ore (with average of 1–1.24 Cu%, conforming to DDH; MDK-11 and MDK-39, Fig. 3a), whereas in marginal areas affected by intensive sericitic overprinting, where potassic alteration is difficult to detect, the enrichment process is incomplete and almost only progressed to form dark halo around pyrite as dominant sulphide and copper grades showed depletion (with average of 0.09–0.28 Cu%, conforming to DDH; MDK-34 and MDK-38, Fig. 3a) rather than hypogene ore (Fig. 3a,b). In the studied profile, based on some potassic intervals with lowest overprinting alteration, average grade of 0.69% approximated for early copper mineralization which is equal to estimated copper average of the whole deposit.

As Brimhall^6^ points out these changes must be according to the destructive effect of acid-oxidant overprinting alteration fluid on buffer minerals of potassic protore, where buffer potential of the rock is preserved copper precipitation and where it totally eliminated almost complete leaching of copper happened. Especially to the east far from center in the marginal areas there are likely deep passages and faults, which have caused penetration of acidic fluid to great depths, relatively severe overprinting on the primary potassic alteration, and also leaching copper out of the parental rock. Scarce presence of turquoise mineral at margins of deposit down to a depth of 600 m indicates infiltration of meteoric water and function of supergenic process in this area (Fig. 3b). Sillitoe^1^ mentioned that late stage overprinting alteration can lead to partial or complete depletion of copper and gold, but metallic concentration may also result, and where intense chlorite-sericitic or sericitic overprinting alteration extended, reconstruction of potassic alteration^1,8^ can lead to complete depletion of original metallic content^1^. Similarly, hypogene oxidation of biotite and magnetite in the rock is proportional to sericitic overprinting intensity, more amount of sericite more oxidizing in the rock happened, insofar magnetic absorption is eliminated by complete conversion to hematite. It should be noted that lithology in the studied profile is almost the same and texture changes are minute, thus this fact rejects lithological control on alteration and enrichment events.

In the studied profile, in low-grade ore at eastern margin of the pit, similar to copper, molybdenum shows the lowest grades as well (with average of 15 ppm, unpublished data). Although based on observations in this study there is common association of copper sulfides with molybdenite (mostly as individual grains) in sericitic halo of veins, the maximum fixation of these two elements in HE event follows different patterns. Generally, Mo content in Miduk is low, but in comparison to maximum abundance of copper sulfides in center, the highest abundance of Molybdenite (with average of 80–105 ppm) matches with sericite-chlorite overprinting, between central and marginal areas.

Such hypogene enrichment evidences in PCDs could indicate their more complex evolutionary pattern and most likely related to polyphasal intrusion emplacement and resultant repeated Cu-Au mineralization^10^, as well as supplying a magmatic source for temperature and sulfur vapor^4,6^. Whereas polyphasal intrusion at Miduk isn’t well known, a cylindrical low grade late/post? stock recently identified located in the center to northwest of the pit (Supplementary, Fig. S14). By drilling proved this stock vertically has extended to the deepest part of the deposit. Presence such intrusions could role play as an appropriate thermal engine for later evolutionary processes.

At Miduk deposit, due to the pervasive presence of bornite obviously as a replacement product, it is possible that formation of bornite mainly be secondary hypogene as a result of enrichment. According to that chalcopyrite is the principle hypogene copper bearing sulphide mineral in Miduk and other PCDs^46^ and typically secondary hypogene formation of bornite in Miduk as an evidence deposit, it is interesting to consider this possibility that formation of bornite in porphyry copper deposits could be mainly attributed to evolutionary processes and as a result of leaching-recycling-fixation or addition copper to the system. With such an assumption cannot be expected in younger deposits with a simpler evolutionary history, the presence of bornite as an important part of mineralization. Braxton et al.^47^, introduces chalcopyrite in Boyongan and Bayugo deposits as only important hypogene copper sulphide phase in the younger diorite stock related to intermineralization stage and diorite porphyry dykes of late mineralization, where bornite is largely absent. Besides bornite, a part of chalcopyrite in Miduk deposit apparently is a replacement product of pyrite in hypogene enrichment processes even at deepest levels.

## Methods

This study fundamentally carried out based on the data extracted from relogging of drill cores compiling with related geochemical data, interpretation of microscopic observations, and ultimately analyzing the results. Due to high geochemical anomalies of copper and sometimes molybdenum with increasing depth at Miduk deposit this study focused on every visible change. According to geochemical data variation, and to obtain comparable mineralization-alteration information both horizontally and vertically, study along an east–west profile including 7 diamond drill holes at the center of the pit covering the highest to lowest Cu grades planned (Fig. 3 and Supplementary, Fig. S1).

### Macroscopic studies

Relogging study at NICICO drill core warehouse carried out for about 6800 m of drill core, providing details including alteration, mineralogy, lithology, vein-veinlet types. In order to determine characteristics of high-grade mineralization and its formation processes, samples with enrichment evidence are considered in macroscopic and microscopic scales. General alteration considered for each box but mineralogy and overprinting features recorded for every change.

### Geochemical data

Copper grades used in this research (around 3400 sample data) analyzed by X-ray fluorescence spectrometer (XRF) and inductively coupled plasma-optical emission spectrometer (ICP-OES) instruments at NICICO. Every 2 m of drill cores are cut in half for each sample analysis. Samples crushed and grinded to a fine particle size of 75 µ. The XRF analysis was performed by a Philips PW 1480 instrument. Sample powder mixed with boric acid binder (HBO_3_, binder to sample powder portion applied as 1:3), pressed powder pellets prepared by Herzog automatic hydraulic press. The ICP-OES analysis was performed by an Agilent 5110 instrument. In detail, a mixture of 0.5 gr of sample powder with 20 mL of Aqua Regia (5 mL HNO_3_ + 15 mL HCl) boiled, completely dried and burned over hitter. Burned mass completely diluted with addition of 5 mL HCl and 10–15 mL H2O and measured at a specific final volume of 100 mL.

### Microscopic studying

550 selected samples prepared as polished uncovered thin sections (30 µm thickness). Microscopic studying for all the polished-thin sections in both reflected and transmitted lights and digital imaging done at NICICO using Leica DMLP and Leica DFC280 instrument facilities, respectively. IMA^48^ and IMA-CNMNC^49^ mineral symbols used for mineral abbreviation in figures.

### SEM-BSE-EDX

Following optical microscopy, for more detail consideration of parent-product sulphide boundaries SEM-BSE (back-scatter electron) images and SEM–EDX (energy dispersive X-ray) analysis applied using LEO-1450VP instrument at Ferdowsi University of Mashhad, central Lab. The instrument operated at an accelerating voltage of 20 kV, using W (Agar A054) filament, beam current of 100 µA and spot size of 290 nm for obtaining elemental scan lines, and data processed using INCA Microanalysis Suite software. Thin section surface coated with gold via SC7620 sputter coater (Polaron) for about 30 min.

### XRD analysis

X-ray pattern of one sample obtained for identifying dark halo’s mineralogy using Explorer (GNR) instrument with Dectris detector type, at integration time 3, step 0.01, accelerating voltage 40 kV and beam current of 30 mA, at Ferdowsi University of Mashhad, central Lab.

### Ethical approval

Reprints and permissions information is available at www.nature.com/reprints.

## Supplementary Information

Supplementary Information.

## Data Availability

The geochemical data that support the findings of this study are available from NICICO but restrictions apply to the availability of these data, which were used under license for the current study, and so are not publicly available. Other data generated or analyzed during this study are included in this published article and its supplementary information file.
