# Supplementary material for: Hypogene enrichment in Miduk porphyry copper ore deposit, Iran
Source: Sci Rep. 2022 Nov 9;12:19133. doi: 10.1038/s41598-022-23501-5 (PMC9646824; doi:10.1038/s41598-022-23501-5)
Supplement: Supplementary file 1 — Supplementary Information. [file 41598_2022_23501_MOESM1_ESM.pdf]

Hypogene Enrichment in Miduk Porphyry Copper Ore Deposit, Iran

Leila YousefiSoorani*, Behnam Shafieibafti, Seyed Masoud Homam, Zahra Abbasloo, Hossein TaghizadehZanooghi

*Correspondence to: leila.yousefi.bahar@gmail.com, leila.yousefisoorani@um.ac.ir, ***ORCID: 0000-0003-2078-9953***

Contents

S1 Summary of geology of Miduk PCD

S2 Hypogene enrichment evidences in macroscopic and microscopic scales

- S2.1 Photographs of drill-cores
- S2.2 Microphotographs of polished thin sections

S3 Analytical data

- S3.1 X-ray diffraction (XRD) analysis
- S3.2 Scanning Electron Microscopy with energy dispersive X-ray analysis (SEM-EDX)

S4 Table of reactions

S5 A note on terminology and abbreviations Used

S6 Photographs of late/post low grade stock

References

S1 Summary of geology of Miduk PCD

Miduk porphyry copper deposit located in Kerman Belt, 85 km west of Sarcheshmeh mine as the largest and most known PCD in Iran. The Kerman Belt, located in southeastern Iran, is a NNW SSE elongated mountain belt 500 km long and 100 km wide. It is principally composed of a folded and faulted early Tertiary volcano-sedimentary complex and is bordered to the southwest by a major thrust zone and the Tertiary and Paleozoic sedimentary rocks of the Zagros Mountains[1]. In an Eocene high-K calc-alkaline volcanic arc formed after cessation of subduction of Tethyan oceanic lithosphere at the Zagros suture zone[2,3], extensive mineralization occurred from the Miocene to the Pliocene and produced porphyry- and vein-style mineralization. Large porphyry copper mines in the region include Sarcheshmeh and Miduk[4,5]. A quartz diorite-diorite stock with an approximate 2 km diameter known as the Miduk porphyry generated Miduk PCD (>441 Mt @ 0.69% Cu[6]). The intrusion hosts 90% of the Cu mineralization and is cross-cut by multiple NNE trending dykes called the Miduk fine porphyry. The dykes are of similar composition to the main intrusion and are interpreted as comagmatic[4]. A limited western part of the stock subdivided as early fine-grained mineralized porphyry and introduced as P1[7,8]. Mineralization, dominantly as chalcopyrite, occurred in both stockwork and disseminated types in extent of deposit. Based on our observations alteration at Miduk happened primarily as wide potassic and narrow marginal propylitic zones, followed by various degrees of overprinting firstly by chlorite-sericite, subsequently sericitic and phyllic by increasing overprint intensity. Wherever the overprinting process is weak, the former potassic zone is evident. Pale green sericite zone is observed rarely as a very thin-borderline generally by passing chlorite-sericite zone to sericite-rich zone. Secondary clay-rich zones formed at upper levels which were exposed to supergene enrichment processes. Arabpour[9] estimated depth of supergene enriched zone varies from 50 to 200 m. covellite and chalcocite are the dominant secondary sulfide minerals in the center of the supergene enriched zone which partially overlaps with the potassic alteration[9]. Higher grade central core of the deposit is matched with the previous pit borders (elongated about 1 km) which is developing recently, However, at colored satellite images surficial stock extent is about 2 km (which is considered in schematic hypothetical cross section, Fig. 3c). Location and geological maps provided in Fig. S1.

The age data for the Miduk porphyry include Zircon U-Pb and zircon (U-Th)/He ages of 12.5 Ma that are essentially identical to mineral-whole rock Rb-Sr ages (12.4 ± 0.5 Ma) reported by Hassanzadeh[3,4], also Ar-Ar isochron ages determined as 11.2 ± 0.5 Ma for biotite in potassic alteration assemblages, and 10.8 ± 0.4 Ma for sericite in phyllic alteration zones[4], moreover, Re-Os age of molybdenite (12.23 ± 0.07) obtained by Taghipour[7]. Based on thermochronometry study by McInnes[3] emplacement depth of 2.5 km determined for Miduk deposit.

Structurally, the case study is located in western part of a dextral shear zone (NW-SE), between Rafsanjan strike slip fault from north and Shahr_e_Babak strike slip fault from south. Locally, most of the faults are dextral with a dip-slip component. Dominant trend of faults is NE-SW (Fig. S1). Faults' slope is approximately perpendicular and their inclination is NW. Most of the dykes are almost vertical with north dominant inclination and general trend coinciding joints. In most cases, faults are faulted joints. The mechanism of the fractures is tensional and shearing-tensional by the main N-S trend for fractures with a dip of >70°. Fault-related fractures often extend in depth as wedge fractures. Two major directions of N-W and E-W display the fracture system of the deposit[10]. Fractures at the Miduk deposit are affected by main fractures in the region. Thus, structures related to the studying stock are conformed to regional tension regime[11].

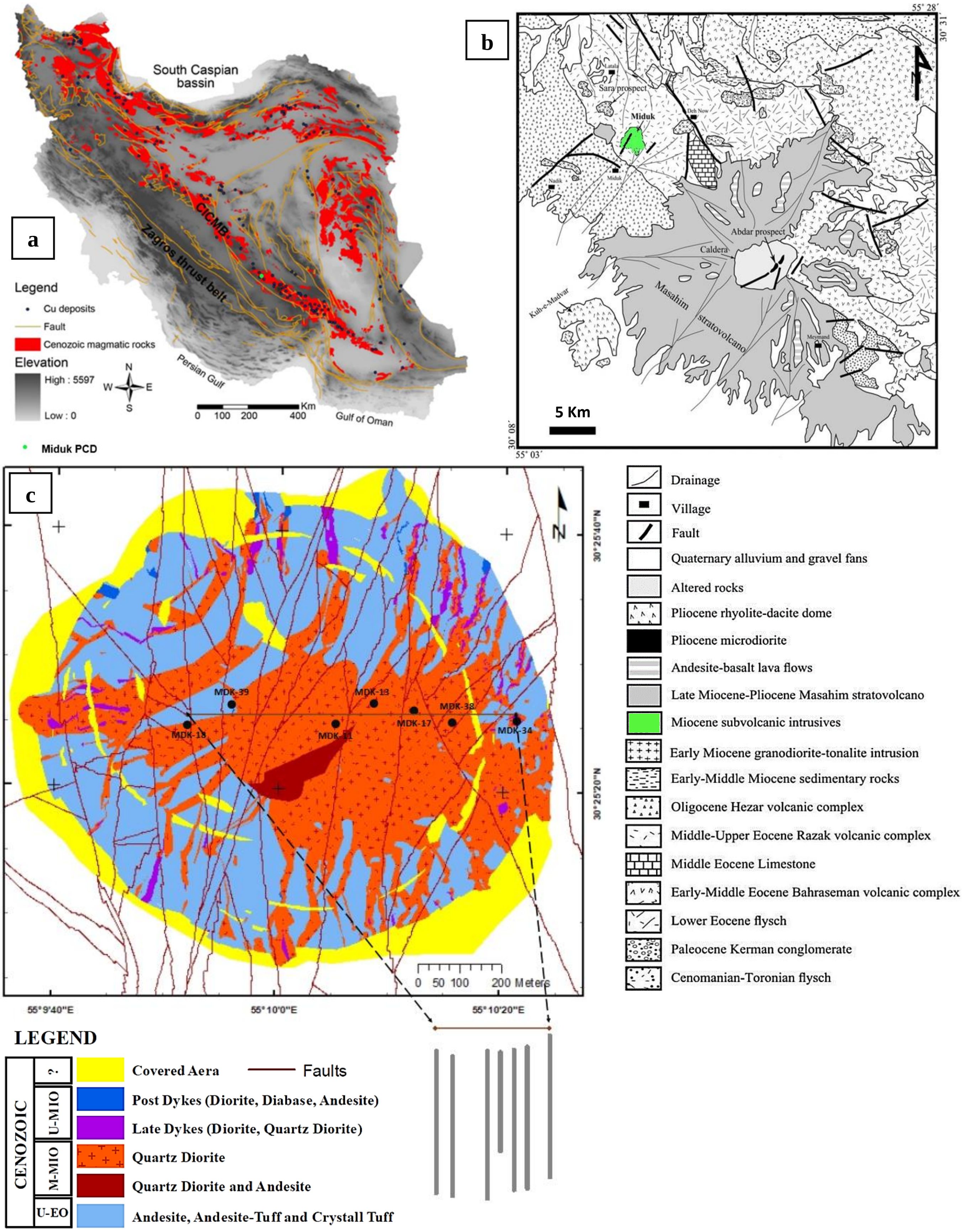

Fig. S1 Locality and geological map of Miduk PCD mine. a position of Miduk PCD in central Iranian Cenozoic magmatic belt (CICMB), map; after Honarmand[12] (https://www.scirp.org/journal/paperinformation.aspx?paperid=71104) **b** Regional geological map modified after Saric et al.[13], Dimitrijevic[14], Taghipour et al.[7]. (https://onlinelibrary.wiley.com/doi/10.1111/j.1751-3928.2008.00054.x) and (http://www.ngdir.ir/advancedSearchContent/1301) **c** Simplified local geological map prepared using ArcGIS 10.5 software (https://desktop.arcgis.com) based on unpublished data from NICICO. Studied profile at the center of deposit including 7 drill core boreholes from ~700 to 1000-meter length.

S2 Hypogene enrichment evidences in macroscopic and microscopic scales

S2.1 Photographs of drill-cores; Figs. S2-S5

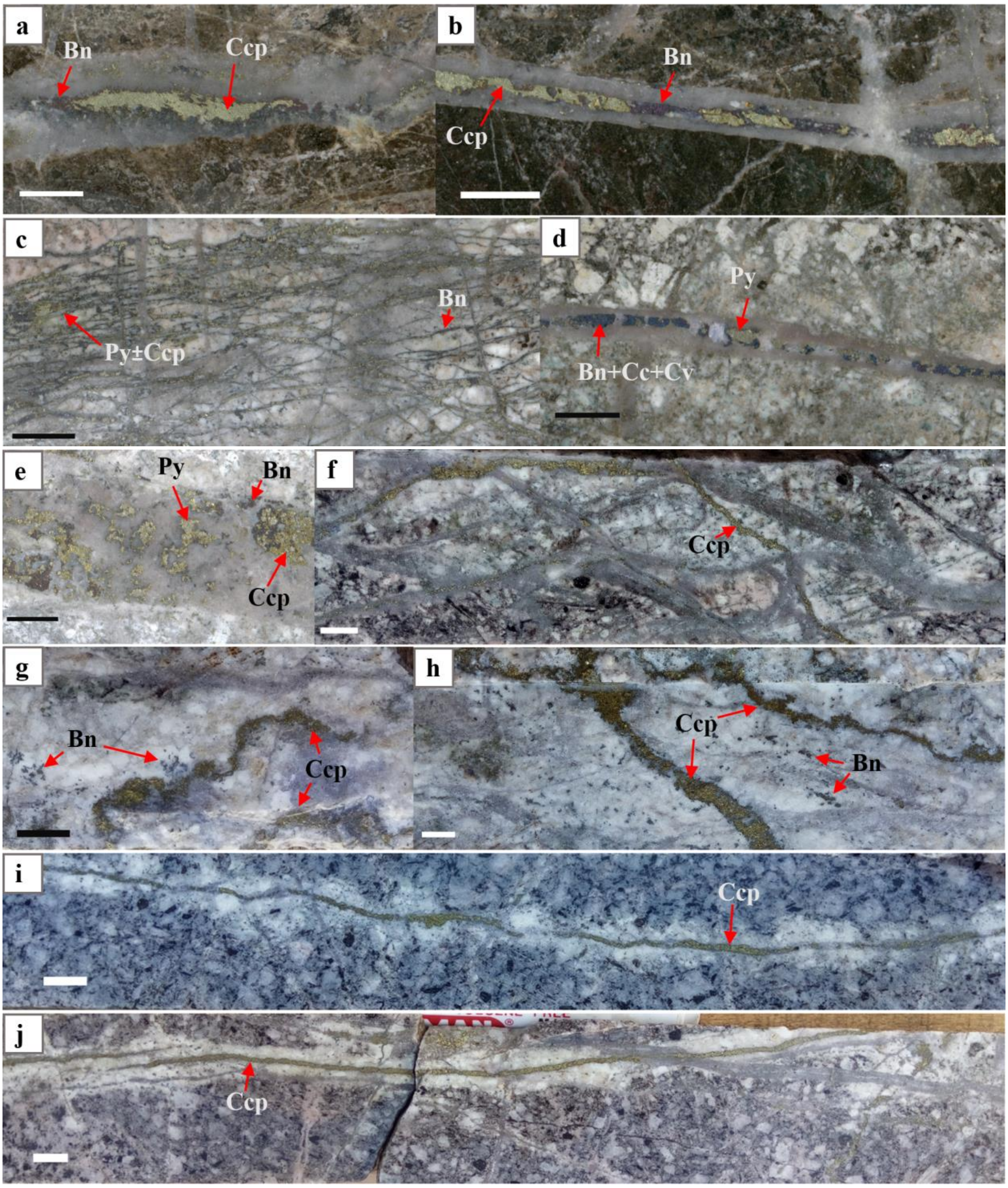

Fig. S2 Photographs of DDH (Diamond Drill Hole) sample pieces with copper-rich mineralized vein/veinlets. a, b replacement of chalcopyrite to bornite in quartz-sulphide vein. **c** Bunch of hairy pyrite-chalcopyrite-bornite microveinlets invading kspr-dominant potassic alteration. **d** replacing pyrite to bornite-chalcocite-covellite in quartz-sulphide vein. **e** pyrite to chalcopyrite to bornite in quartz-sulphide vein, general alteration is phyllic. **f** curved chalcopyrite veinlet cut potassic veins and changed potassic matrix to sericitic in passing way. **g, h** sinusoidal chalcopyrite veins. bornite grains formed in high sericitized text as disseminated dark spots. **i, j** chalcopyrite veinlets with sericitic halo crosscut potassic related phases. scale bar is 1 cm. sample depth from collar, in meter: a (861), b (860), c (678), d (322), e (680), f (677), g (822), h (827), i (835) and j (557). Bn (Bornite), Ccp (Chalcopyrite), Cc (Chalcocite), Cv (Covellite), Py (Pyrite).

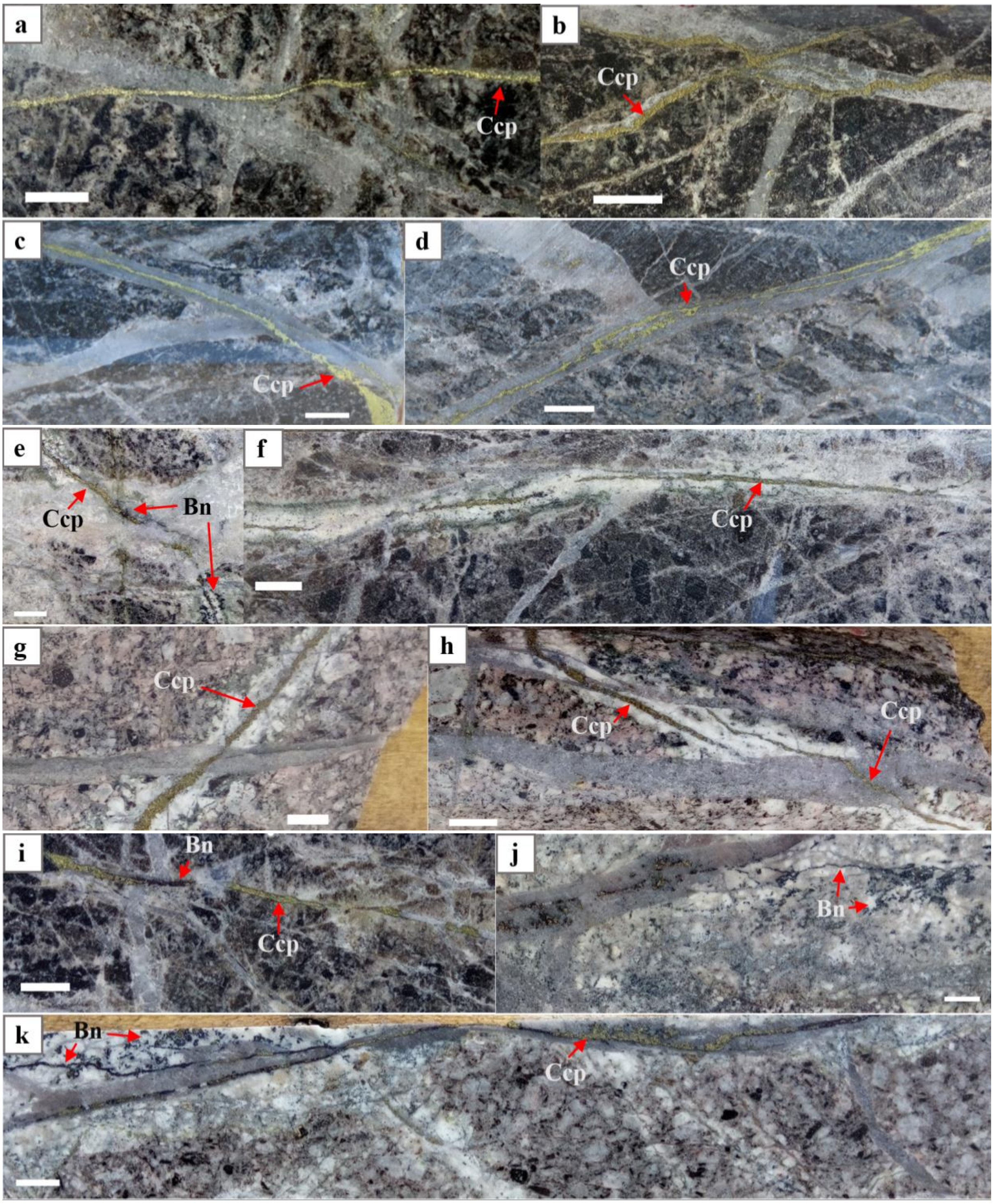

Fig. S3 Photographs of DDH core pieces with copper-rich vein/veinlets. **a-d** potassic vein phases reopened and cut by chalcopyrite veinlets. Later injection of chalcopyrite-forming fluid into quartz-dominant vein is obvious at the margin of the vein as external member entrancing into center. **e-h** chalcopyrite±bornite with sericitic halo cut through text of potassic. There is a gap in sericitic halo formation for intervals where chalcopyrite intrudes into quartz text. chlorite is a common mineral in halo of later sulfide vein/veinlets intruding biotite-rich potassic text. **i** chalcopyrite±bornite±quartz veinlet crosscut potassic phases. **j, k** hairy bornite microveinlets with minute chalcopyrite intruding preformed vein. disseminated bornite grains formed in sericitized context. scale bar is 1 cm. sample depth from collar, in meter: a (900), b (895), c (950), d (950), e (966), f (976), g (631), h (630), i (922), j (713), k (676).

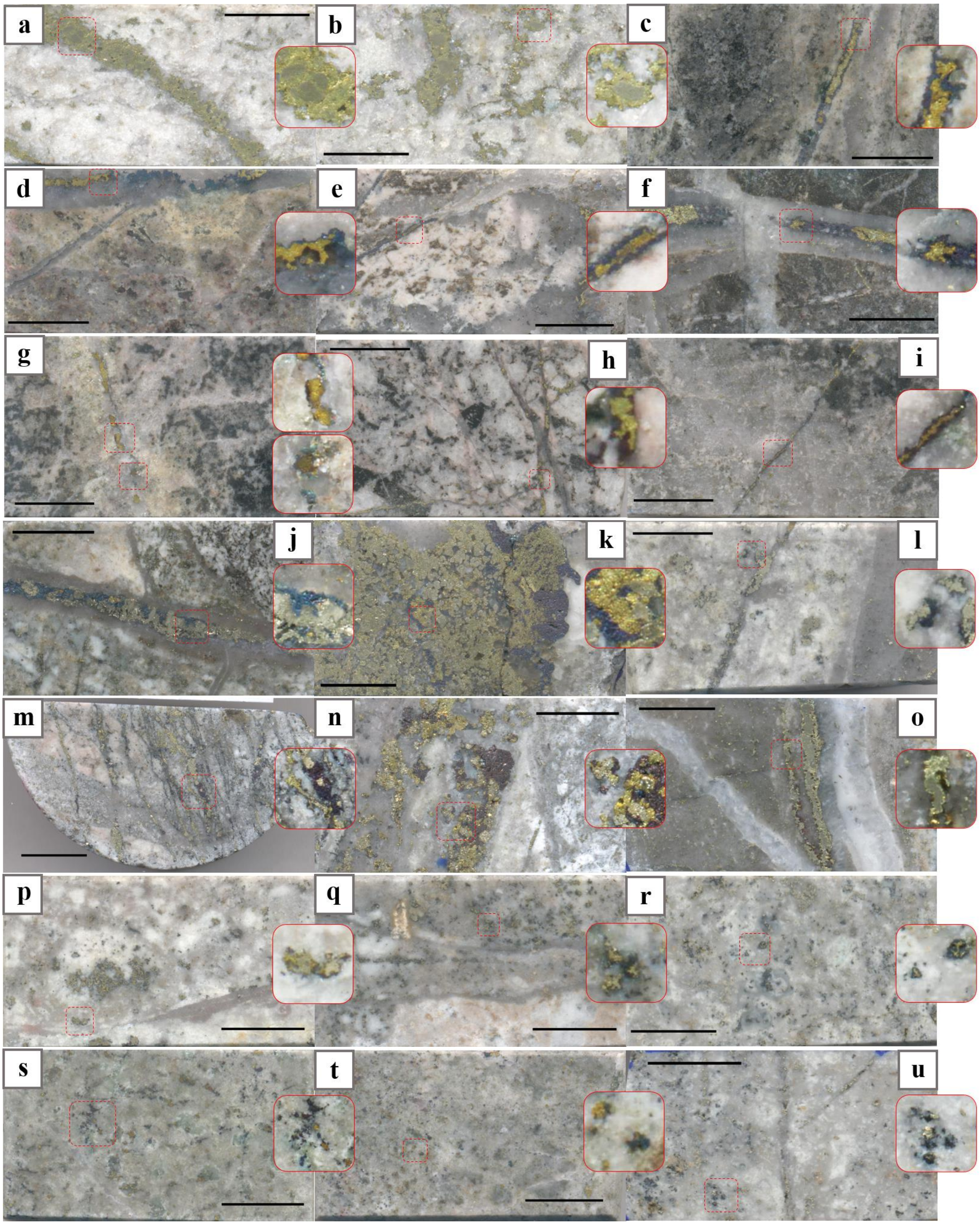

Fig. S4 Photographs of DDH core pieces including sulfide replacement evidences. **a, b** replacement of pyrite by chalcopyrite in high sericitic text. **c-i** replacement of chalcopyrite by bornite in veinlets. transformed chalcopyrite darkened to green and deep violet bornite product at outer surface of chalcopyrite obviously is shown in g. **j** replacement of pyrite to bornite-chalcocite-covellite. **k** pyrite at the core of chalcopyrite changing to bornite. **l** pyrite transformed to bornite as both disseminated and in veinlet. **m, n** replacement of pyrite to chalcopyrite and bornite. **o** narrow rim of chalcopyrite formed at margin of pyrite veinlet. **p-u** replacement of disseminated pyrite/chalcopyrite to bornite. enrichment progress can be seen by forming chalcopyrite after pyrite changing to bornite in p. r and u are related to supergene level. scale bar is 1 cm. sample depth from collar, in meter: a (827), b (1001), c (414), d (861), e (862), f (860), g (944), h (891), i (949), j (322), k (430), l (212), m (678), n (680), o (529), p (417), q (234), r (58), s (886), t (1000) and u (58).

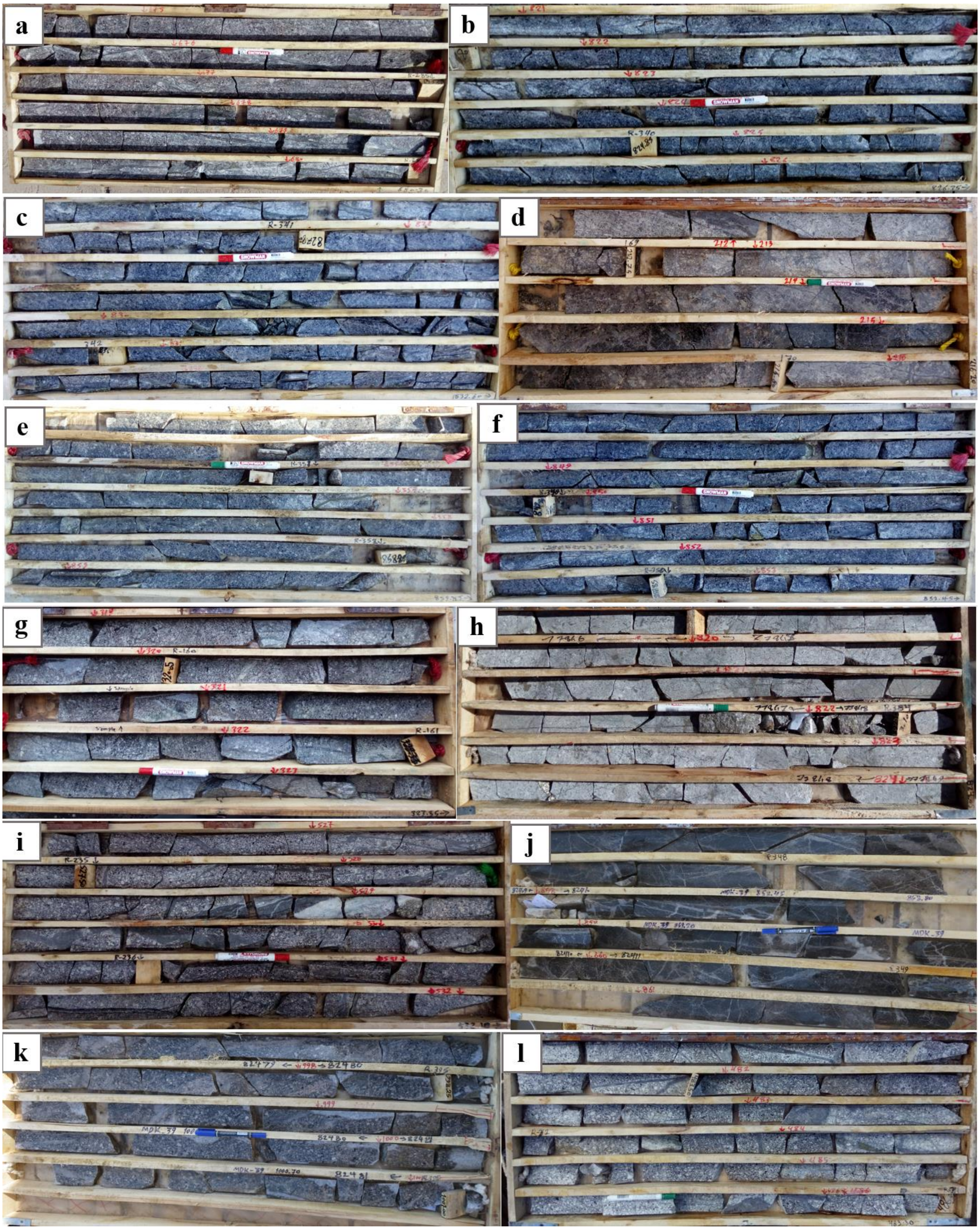

Fig. S5 General alteration in some drill-core boxes. General alteration in all these boxes is potassic in spite of one (h) which shows deep hypogene phyllic at eastern side of the studied profile. Each selected box is related to a specific image from previous provided figures. **a** (Fig. S2-e) **b** (Fig. S2-g) **c** (Fig. S4-a) **d** (Fig. S4-p) **e** (Fig. S6-a) **f** (Fig. S6-m) **g** (Fig. S7-e) **h** (Fig. S7-f) **i** (Fig. S9-d) **j** (Fig. S9-e) **k** (Fig. S9-k) **l** (Fig. S10-d).

S2.2 Microphotographs of polished thin sections; Figs. S6-S10

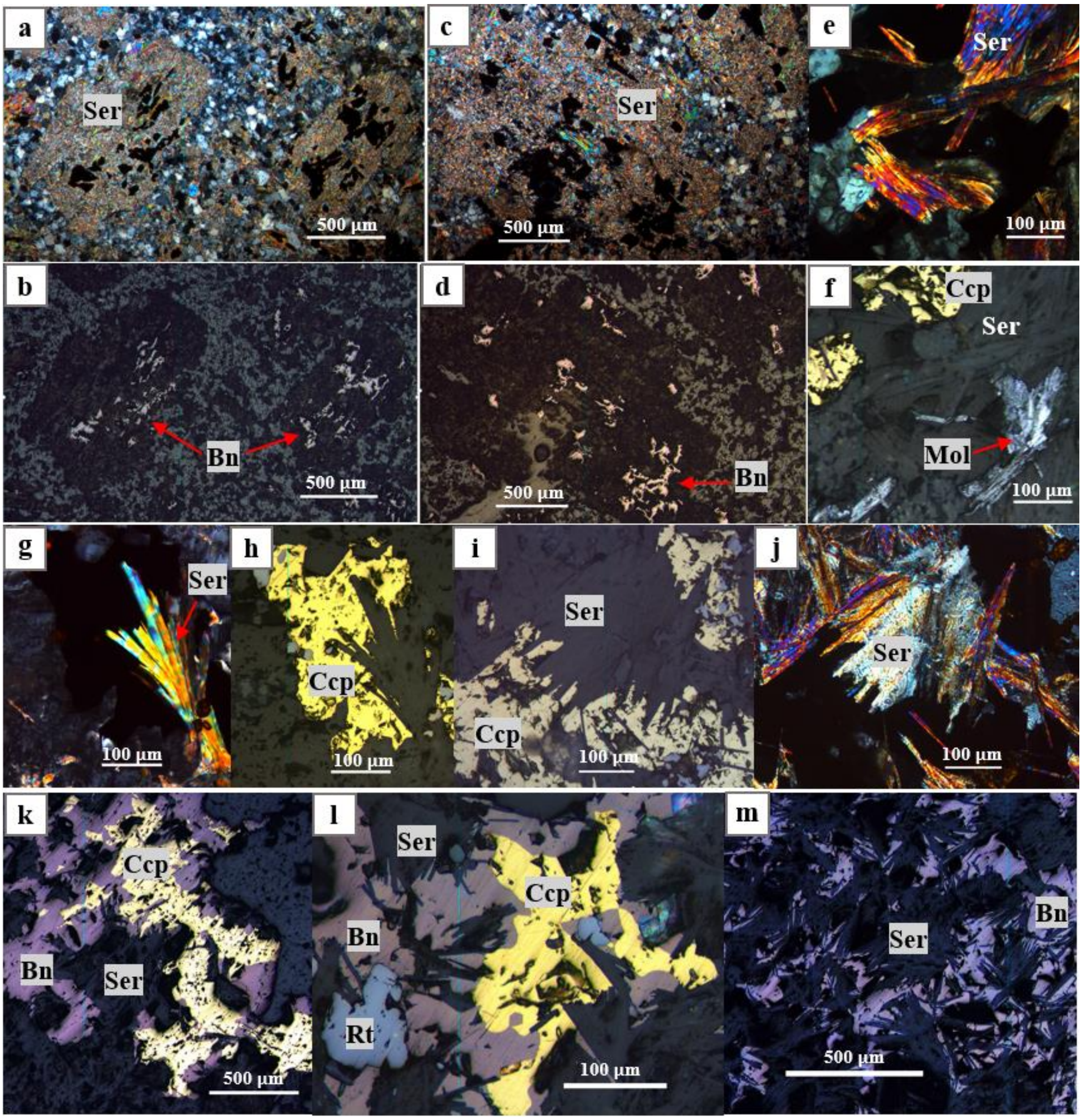

Fig. S6 Reflected and transmitted light microscopic photographs of copper mineralization in close association with sericite (Ser). a-d phenocrysts entirely replaced by sericite and bornite. **e-j** chalcopyrite± molybdenite (Mol) intergrowth with sericite/muscovite blades. **k-m** intergrowth of bornite /+ chalcopyrite with sericite/muscovite, intergrowth of sericite blades caused sharp straight boundaries between chalcopyrite transformed to bornite. Rutile (Rt) is a common byproduct of replacement events. sample depth from collar, in meter: a-d (857), e-h (558), i, j (944), k (740), l (258) and m (851).

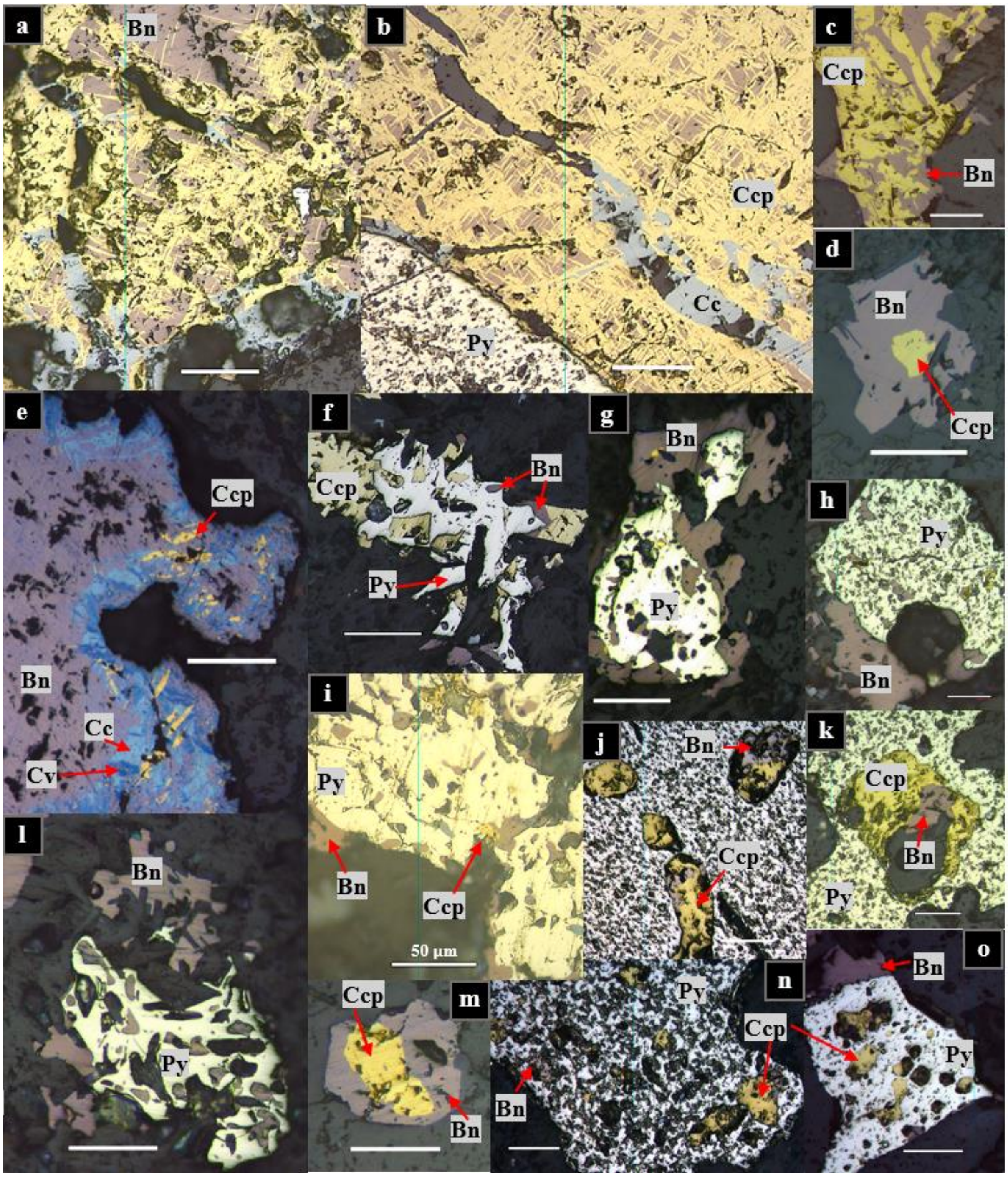

Fig. S7 Reflected light microscopic photographs of sulfide replacement textures. **a, b** exsolution-like replacement texture of chalcopyrite to bornite. Chalcocite formed as veinlets or marginal replacement. **c** chalcopyrite replacement by bornite. **d** consistent bornite-chalcopyrite boundary with outer rim of the grain reflects monotonous replacement front. **e** bornite replaced by chalcocite-covellite±chalcopyrite assemblage at outer surface. Chalcopyrite in retrograde reaction exsolved from bornite associated with product minerals. **f-o** replacement as inclusion texture of bornite/chalcopyrite in pyrite grains. **m** shows a chalcopyrite single grain transformed to bornite in quartz matrix of vein. The scale bar is 100 µm, despite i. sample depth from collar, in meter: a, b (692), c (1001), d (543), e (322), f (821), g (365), h (436), i (234), j (953), k, l (436), m (645), n (528) and o (258).

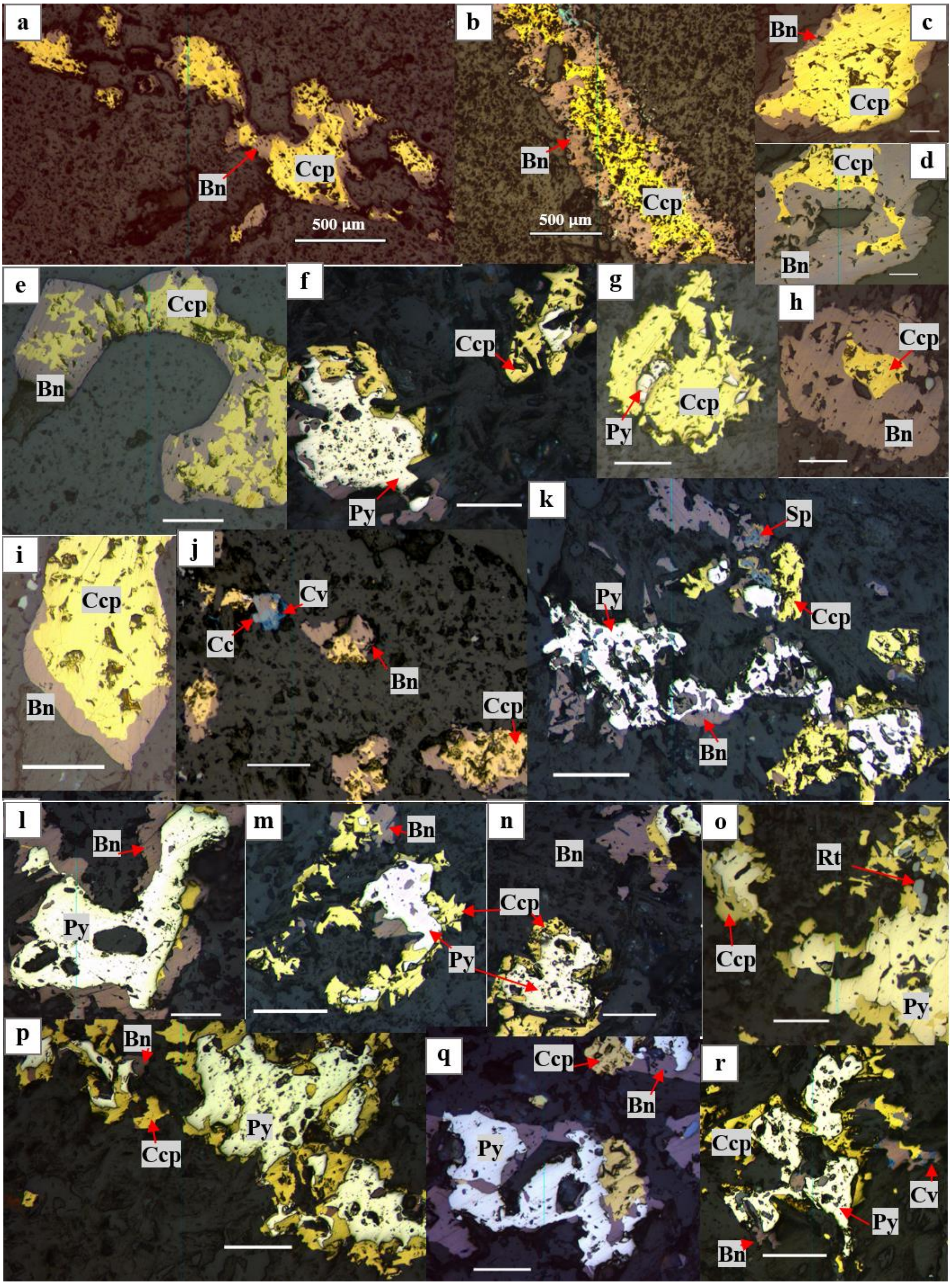

Fig. S8 Reflected light microscopic photographs of rimmed replacement texture of sulfides. **a-e** bornite replaced chalcopyrite from the outer rim of veinlets. **f** porous pyrite replaced to chalcopyrite/bornite from outer rim and also inside pores. **g** remnant texture of pyrite replaced by chalcopyrite. **h** remnant chalcopyrite core after replacing to bornite. **i** consistent bornite-chalcopyrite boundary with outer rim of grain shows monotonous replacement front. **j** chalcopyrite disseminated grains replaced by bornite. A single bornite grain with remnants of chalcopyrite replaced to chalcocite-covellite. **k** pyrite grains are replaced to chalcopyrite/bornite and in some cases to sphalerite (Sp). **l** remnants of chalcopyrite in reaction front of pyrite to bornite. **m-r** pyrite grains replaced by chalcopyrite/bornite from outer rim. Minute covellite formed after bornite (r). progressive copper enrichment can be addressed by triple replacement of pyrite to chalcopyrite to bornite to covellite. advance in replacement of chalcopyrite to bornite comparable in c, d, h. scale bar is 100 µm, despite a, b. sample depth from collar, in meter: a-d (891), e (645), f (258), g (275), h (959), i (645), j (345), k (275), l (365), m, n (275), o (586), p (440), q (365) and r (440).

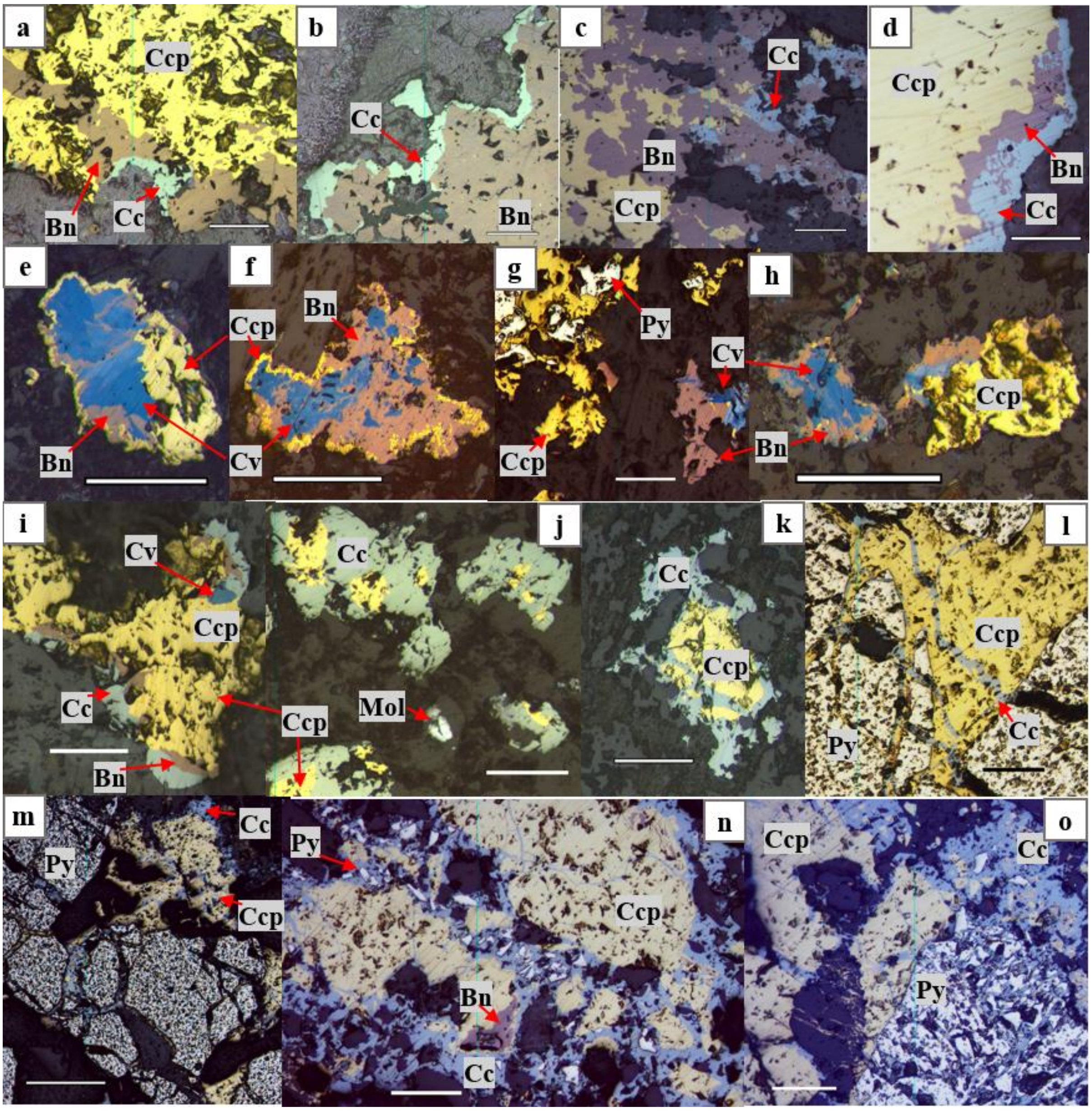

Fig. S9 Reflected light microscopic photographs of copper enrichment. **a-d** progressive copper enrichment event at outer surfaces by replacing bornite product after chalcopyrite to chalcocite. **e-h** bornite after chalcopyrite replaced to covellite. chalcopyrite lamellae retrogressively exsolved from replaced product at outer rim of bornite (bornite oxidation and back replacement[15,16]). **i-k** chalcopyrite replaced to chalcocite±bornite/covellite. **l-o** in most cases, pyrite in veins is brecciated and spaces generally filled by fluid forming chalcopyrite/chalcocite. Chalcopyrite replaced by chalcocite±bornite. The scale bar is 100 µm. sample depth from collar, in meter: a, b (860), c (861), d, i (529), e, f, h (861), g (440), j (606), k (1001) and l-o (692).

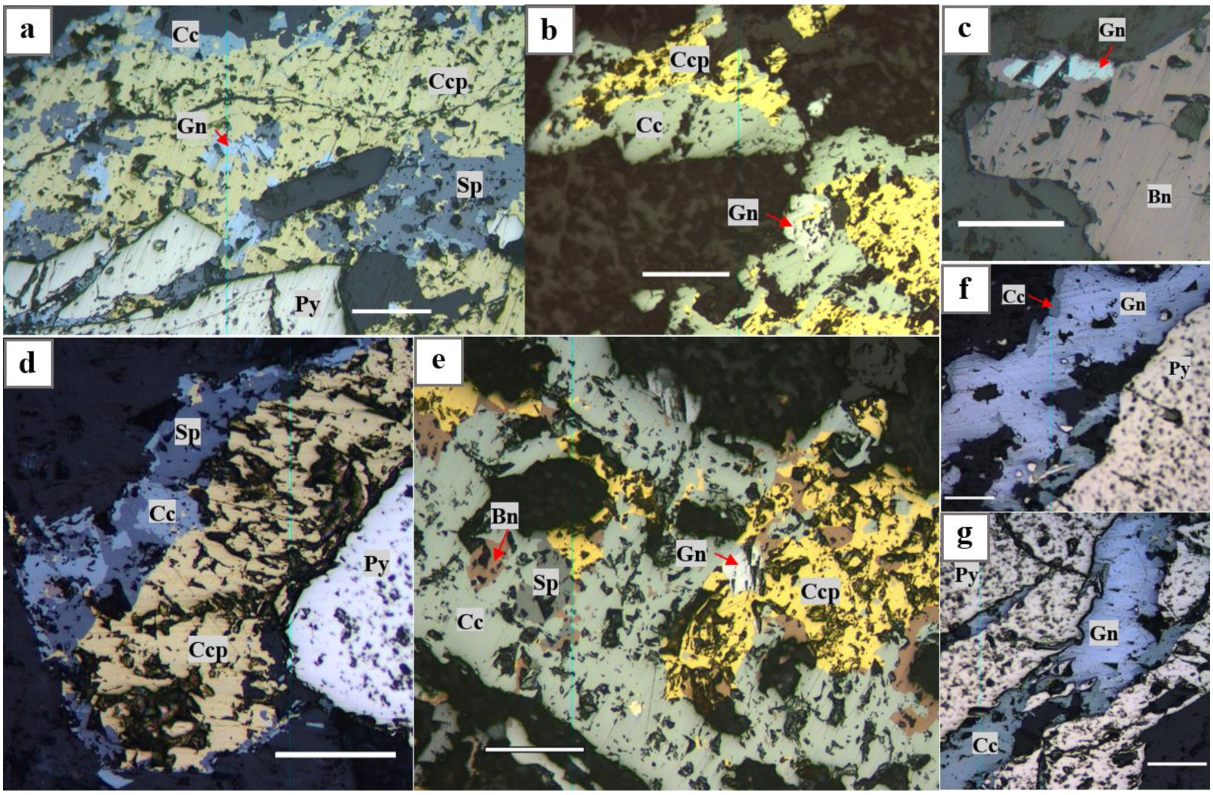

Fig. S10 Reflected light microscopic photographs of polymetallic assemblage associated copper enrichment. Although polymetallic assemblage mostly observed at intermediate levels, also comes into view down to 800-meter level. brecciated pyrite filled by assemblage of minerals including chalcopyrite, chalcocite, galena (Gn) and sphalerite. **b** outer surfaces of chalcopyrite replaced by chalcocite and galena. **c** bornite at outer rim chemically changed to galena. **d** sphalerite-chalcocite replaced chalcopyrite. chalcopyrite overgrowth/replaced pyrite. **e** polymetallic vein including chalcopyrite-chalcocite-bornite-galena-sphalerite. **f, g** galena-chalcocite overgrowth/filled pyrite in vein structure. The scale bar is 100 µm. sample depth from collar, in meter: a (722), b (606), c (987), d (483), e (414) and f, g (442).

S3 Analytical data

S3.1 X-ray diffraction (XRD) analysis; Fig. S11

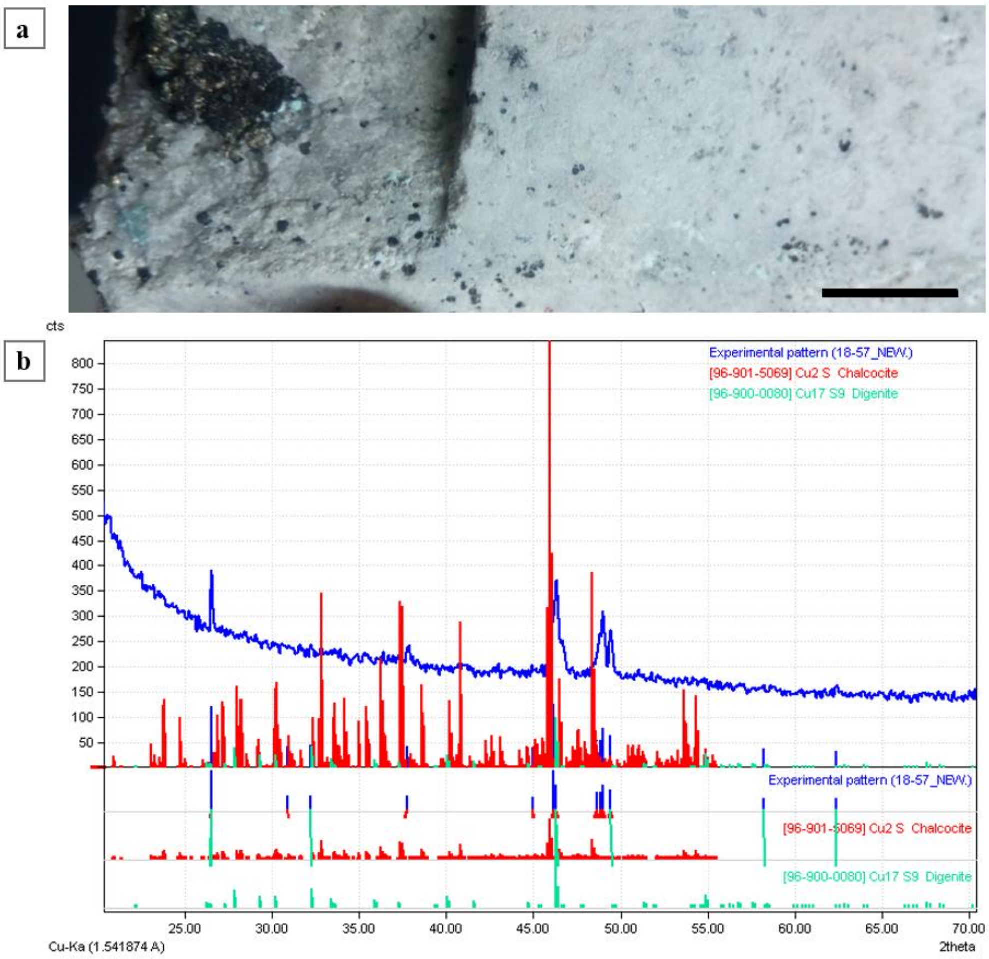

Fig. S11 a Photograph of earthy luster dark halo around pyrite grains in context of phyllic alteration. The sample belongs to supergene level. The pale blue fine grain of secondary copper ore mineral associated with pyrite and chalcocite is probably immature turquoise. Scale bar is 1 cm. **b** XRD spectral pattern of the dark halo indicates related mineralogy as chalcocite and digenite.

S3.2 Scanning Electron Microscopy with energy dispersive X-ray analysis (SEM-EDX); Figs. S12, S13

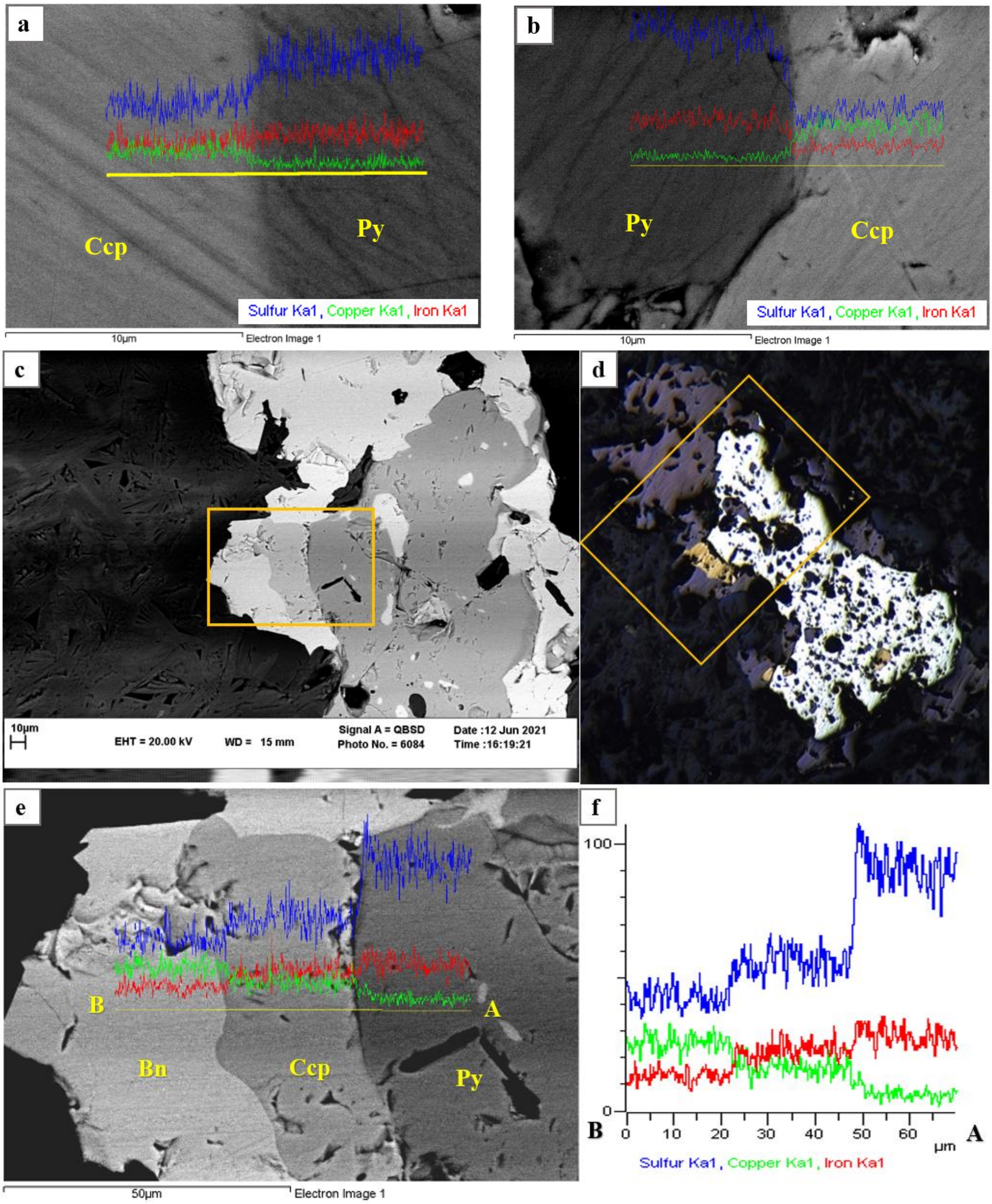

Fig. S12 SEM-EDS analysis scan lines illustrate gradual copper-iron-sulfur variations in borders between sulfide phases confirming replacement in disseminated ore grain. a, b SEM-EDS analysis micrographs on Back-scatter electron (BSE) photomicrograph shows smooth and gradual increasing in copper content in the border passing from pyrite to chalcopyrite. **c, d** BSE image and reflected light of a pyrite grain replacing to chalcopyrite and bornite. **e, f** BSE-EDS along the scan line from A to B also shows no sharp increase in copper content in pyrite- chalcopyrite and chalcopyrite -bornite borders. Sample; DDH: MDK-13 depth from collar: 258 meters.

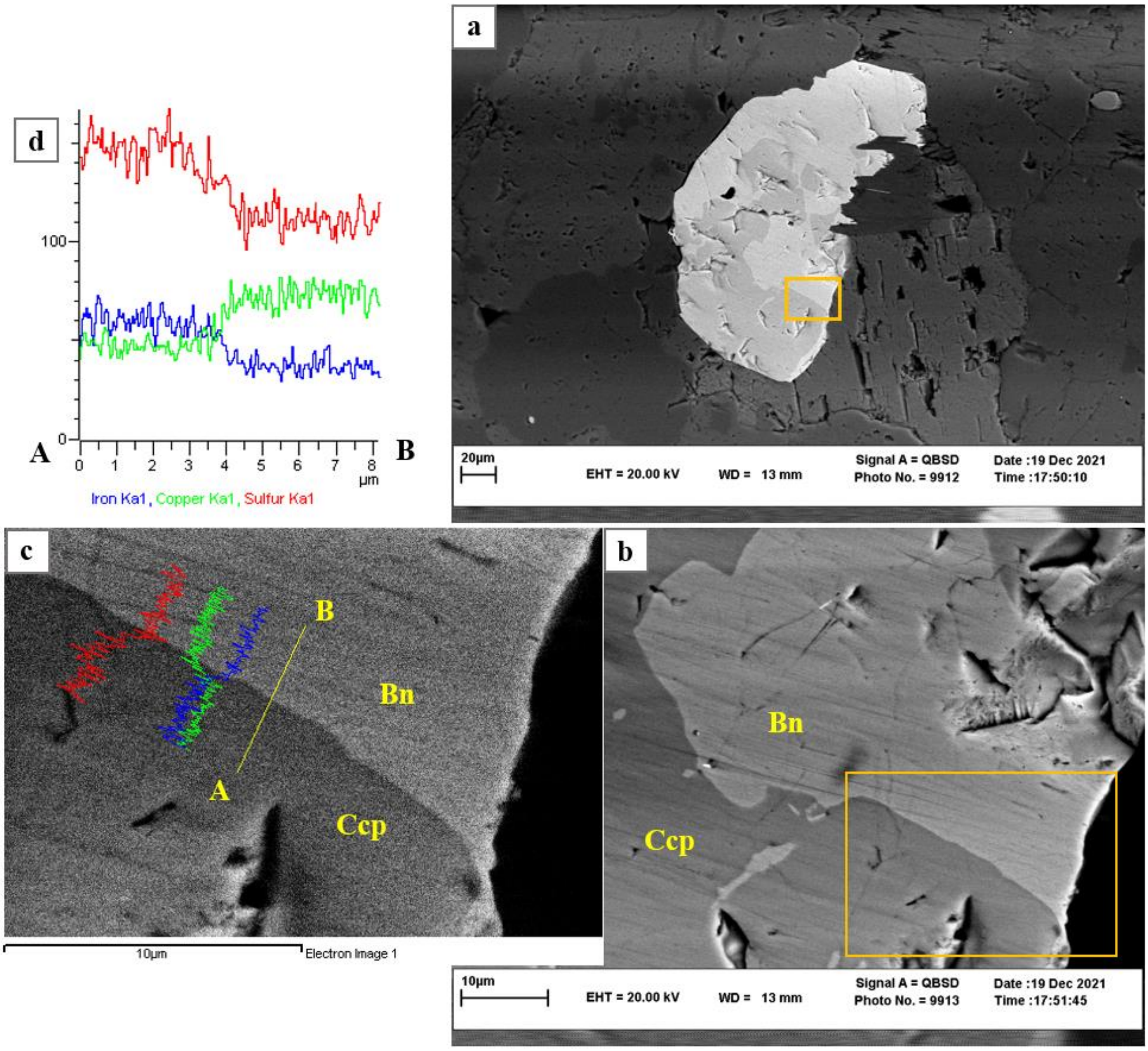

Fig. S13 Replacement of chalcopyrite by bornite in a disseminated ore grain. a, b BSE images of single disseminated chalcopyrite grain replaced by bornite. **c, d** EDS analysis micrographs shows smooth and gradual increasing in copper content in the border passing from chalcopyrite to bornite. sample; DDH: MDK-11 depth from collar: 977 meters.

S4 Table of reactions

Table S1 Theoretical and experimental laboratory equations related copper-iron sulfide replacements.

Replacement Phases	Formula of Reaction	Reference
**Ccp to Bn**	$CuFeS_{2(s)} + 4Cu(HS)_2 = Cu_5FeS_{4(s)} + 6\ HS^- + 2H_2O$	Zhao et al.[17]
**Ccp to Bn**	$2CuFeS_{2(s)} + 3Cu(HS)_2 + 3OH^- = Cu_5FeS_{4(s)} + Fe(OH)_{2(aq)} + 6HS^- + 0.5H_2O + 0.25O_{2(g)}$	Zhao et al.[17]
**Ccp to Bn**	Chalcopyrite + 2 Chalcocite = Bornite: ($CuFeS_2 + 2Cu_2S = Cu_5FeS_4$)	Zhao et al.[17]
**Ccp to Bn**	$CuFeS_2 + OH^- + 1.5H_2O = Cu(HS)_2^- + Fe(OH)_{2(aq)} + 0.75O_{2(aq)}$<br>$5Cu(HS)_2^- + Fe(OH)_{2(aq)} + OH^- + 0.25O_{2(aq)} = Cu_5FeS_{4(s)} + 6HS^- + 3.5\ H_2O$	Zhao et al.[17]
**Ccp to Bn**	$CuFeS_{2(s)} + 2H_2S_{(aq)} + 4CuCl_2^- = Cu_5FeS_{4(s)} + 4HCl_{(aq)}$	Zhao et al.[18]
**Ccp to Bn**	$CuFeS_2 = Cu^{2+} + Fe^{2+} + S^0 + 4e^-$<br>$CuFeS_2 + 8H_2O = Cu^{2+} + Fe^{2+} + 2SO_4^{2-} + 16H^+ + 16e^-$<br>$CuFeS_2 + 8H_2O = Cu^{2+} + Fe^{2+} + 2HSO_4^- + 14H^+ + 16e^-$<br>$2CuFeS_2 + 3Cu^{2+} = Cu_5FeS_4 + Fe^{2+}$<br>$5Cu^{2+} + Fe^{2+} + 4SO_4^{2-} + 32H^+ + 16e^- = Cu_5FeS_4 + 16H_2O$	Sikka et al.[19]
**Ccp to Bn**	$CuFeS_2+2Cu_2S = Cu_5FeS_4$	Amcoff[20]
**Py to Ccp**	$FeS_2 + 4H^+ + 2Cl^- + 2e^- = FeCl_{2(aq)} + 2H_2S_{(aq)}$<br>$CuCl_2^- + FeCl_{2(aq)} + 2H_2S_{(aq)} = CuFeS_2 + e^- + 4Cl^- + 4H^+$	Zhang et al.[21]
**Py to Ccp**	$FeS_2 + 14/\ 15CuCl_2^- + 8/\ 15H_2O = 14/\ 15CuFeS_2 + 14/\ 15H^+ + 1/\ 15FeCl_{2(aq)} + 2/\ 15\ HSO_4^- + 26/\ 15Cl^-$	Zhang et al.[21]
**Py to Cc**	$5FeS_2 + 14Cu^{2+} + 12H_2O = 7Cu_2S + 5Fe^{2+} + 24H^+ + 3SO_4^{2-}$	Schumer et al.[22]
**Ccp to Cc**	$5CuFeS_2 + 11Cu^{2+} + 12H_2O = 2Cu_2S + 5Fe^{2+} + 24H^+ + 3SO_4^{2-}$	Schumer et al.[22]
**Py to Bn**	$2FeS_2 + 5Cu_2S + OH- = 2Cu_5FeS_4 + HS-+ 0.5O_2(g)$<br>$2FeS_2 + 5Cu_2S + H_2O = 2Cu_5FeS_4 + H_2S+ 0.5O_2(g)$<br>$FeS_2 + 5Cu(HS)_2 + 0.5O_2(g) = Cu_5FeS_4 + 8HS^- + H_2O$	This study
**Ccp to Bn**	$5CuFeS_2 + 6H_2O = Cu_5FeS_4 + 2Fe_2O_3 + 6H_2S$<br>$2CuFeS_2 + 3Cu^{2+} + 0.75O_2 = Cu_5FeS_4 + 0.5Fe_2O_3$	This study

S5 A note on terminology and abbreviations Used

Active pyrite; a technical phrase in flotation, mineral processing, referring to pyrite partly to entirely coated by or joined to copper sulfides specifically chalcocite, lead to attract the grain by bobbles of collector material and move into the concentrate.

Back replacement texture; generally, parent phase replaces by product in forward reaction, inversely the product phase replaces by parent phase in retrograde state, eg. back replacement of bornite by chalcopyrite[15,16,23].

Collar; the mouth of a drilling bore hole, the target point which drilling equipment set up at and drill bit started.

HE; Hypogene Enrichment, MDK; Miduk, PCD; Porphyry Copper Deposit, Bn; Bornite, Py; Pyrite, Ccp; Chalcopyrite, Cv; Covellite, Cc; Chalcocite, Sp; Sphalerite, Gn; Galena, Tqu; Turquoise, Ser; Sericite, Mol; Molybdenite, Hem; Hematite, Rt; Rutile, Pts; Potassic, Sup; Supergene, QSP; Quartz-Sericite-Pyrite (Phyllic), Chl-Ser; Chlorite-Sericite, DDH; Diamond Drill Hole, NICICO; National Iranian Copper Industries Company.

S6 Photographs of late/post low grade stock; Fig. S14

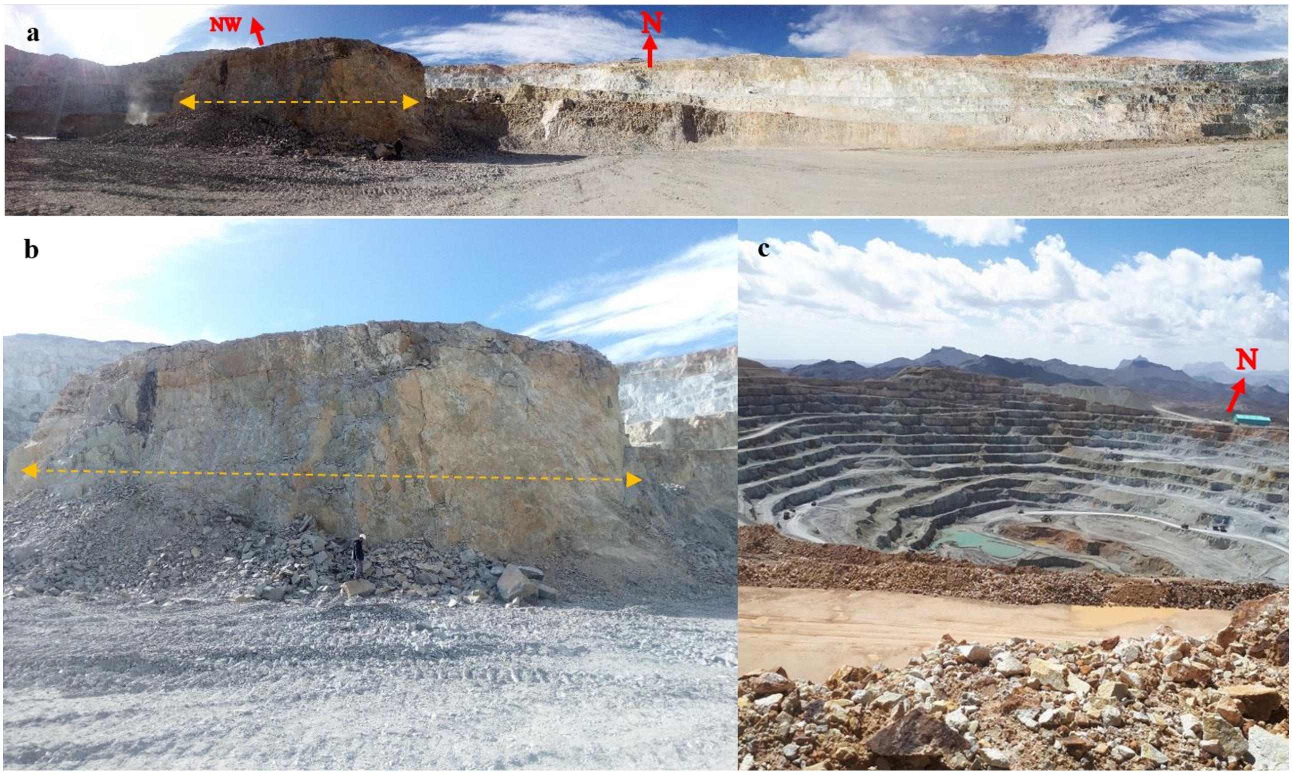

Fig. S14 Photographs of the low grade stock in the Miduk mine pit. a, b the cylindrical low-grade late/post? stock at Miduk pit bottom, a; panorama photo. Dashed orange line indicating the stock dimension. The one as scale is around 170 cm. **c** open pit of Miduk mine, sight to northwest. The date of photograph c is different from a, b.

References:

1. Waterman, G.C. and Hamilton, R.L. The SarCheshmeh porphyry copper deposit. *Econ. Geol.* **70**, 568-576 (1975).
2. Sengor, A.M.C. and Kidd, W.S.F. Post-collisional tectonics of the Turkish-Iranian plateau and a comparison with Tibet. *Tectonophysics* **55**, 361–376 (1979).
3. McInnes, B.I.A., Evans, N.J., Fu, F.Q., Garwin, S., Belousova, E., Griffi n, W.L., Bertens, A., Sukarna, D., Permanadewi, S., Andrew, R.L., Deckart, K. Thermal history analysis of selected Chilean, Indonesian and Iranian porphyry Cu –Mo –Au deposits. In: Porter, T.M. (Ed.), Super porphyry Copper and Gold Deposits — a Global Perspective. *Adelaide PGC Publishing*, 27 –42 (2005).
4. Hassanzadeh, J. Metallogenic and tectonomagmatic events in the SE sector of the Cenozoic active continental margin of central Iran (Shahr e Babak area, Kerman Province). Unpublished Ph.D. Thesis, UCLA, 204 (1993).
5. Mars, J.C. Regional mapping of hydrothermally altered igneous rocks along the Urumieh-Dokhtar, Chagai, and Alborz Belts of western Asia using Advanced Spaceborne Thermal Emission and Reflection Radiometer (ASTER) data and Interactive Data Language (IDL) logical operators—A tool for porphyry copper exploration and assessment. U.S. Geological Survey Scientific Investigations Report 2010–5090–O, 36 (2014).
6. NICICO (National Iranian Copper Industries Company). Exploration report of Miduk PCD under exploitation. Unpublished internal report, 247 (2018).
7. Taghipour, N., Aftabi, A., and Mathur, R. Geology and Re-Os Geochronology of Mineralization of the Miduk Porphyry Copper Deposit, Iran. *Resource Geology* **58**, No. 2, 143-160 (2008).
8. Aghazadeh, M., Hou, Z., Badrzadeh, Z., Zhou, L. Temporal–spatial distribution and tectonic setting of porphyry copper deposits in Iran: constraints from zircon U-Pb and molybdenite Re–Os geochronology. *Ore Geol. Rev*. **70**, 385–406 (2015).
9. Arabpour, A., Mirnejad, H., Asghari, O., Moosavi, M. Copper mass flux and evolution of supergene orebody in the Miduk Porphyry Copper Deposit, Iran, and its application to exploration of exotic deposits. *Ore Geol. Rev.* **139** (B), 18 (2021).
10. Ebadi, L. Structural analysis of Miduk (Lacha) mine and its surroundings (northeast of Shahr-e-Babak). M.Sc. thesis (in Persian), Shahid Beheshti University, 104 (2009).
11. Ebadi, L., Alavi, S.A., Shafieibafti, S. Development of tensile structures in a high-pressure regime and their relationship with the formation of vein-type and porphyry copper deposits in the northeast of Shar-e-Babak. (in Persian), *Iranian Journal of Earth Sciences* **81**, 101-114 (2011).
12. Honarmand, M. Application of Airborne Geophysical and ASTER Data for Hydrothermal Alteration Mapping in the Sar-Kuh Porphyry Copper Area, Kerman Province, Iran. *Open Journal of Geology* **6**, 1257-1268 (2016).
13. Saric, A., Djordjevic, M. and Dimitrijevic, M. N. Geological map of Shahr-Babak, Scale 1/100000 Geological Survey of Iran, Tehran, Iran (1971).
14. Dimitrijevic, M. Geology of Kerman region. (Iran Geological Survey Report No. Yu/52.) Institute for Geological and Mining Exploration and Institution of Nuclear and Other Mineral Raw Materials, Beograd-Yugoslavia, 334 (1973).
15. Amcoff, O., Hedin, J.O. Mechanism of formation of composite chalcopyrite-copper sulfide lamellae in bornites from Gruvberget, northern Sweden. *Geologiska Föreningen i Stockholm Förhandlingar*, **113**:1, 15-23 (1991).
16. Li, K., Brugger, J., Pring, A. Exsolution of chalcopyrite from bornite-digenite solid solution: an example of a fluid-driven back-replacement reaction. *Mineral. Deposita* **53**, 903–908 (2018).
17. Zhao, J., Bugger, J., Ngothai, Y., Pring, A. The replacement of chalcopyrite by bornite under hydrothermal conditions. *American Mineralogist* **99**, 2389–2397 (2014).
18. Zhao, J., Brugger, J., Chen, G., Ngothai, Y. and Allan Pring, A. Experimental study of the formation of chalcopyrite and bornite via the sulfidation of hematite: Mineral replacements with a large volume increase. *American Mineralogist* **99**, 343–354 (2014).
19. Sikka, DB., Petruk, W., Nehru, Ch.E., Zhang, Zh. Geochemistry of secondary copper minerals from Proterozoic porphyry copper deposit, Malanjkhand, India. *Ore Geol. Rev.* **6**, 257-290 (1991).
20. Amcoff, O. Experimental replacement of chalcopyrite by bornite: textural and chemical changes during a solid-state process. *Mineral. deposita* **23**, 286-292 (1988).

21. Zhang, Y., Cai, Y., Qu, Y., Wang, Q., Gu, L., Li, G. Two-stage fluid pathways generated by volume expansion reactions: insights from the replacement of pyrite by chalcopyrite. *Nature Research* **10**: 19993 (2020).
22. Schumer, B.N., Stegen, R.J., Barton, M.D., Hiskey, J.B., Downs, R.T. Mineralogical profile of supergene sulfide ore in the Western copper area, Morenci mine, Arizona. *The Canadian Mineralogist* **57**, 391-401 (2019).
23. Roberts, W.M.B. The low temperature synthesis in aqueous solution of chalcopyrite and bornite. *Ecol. Geol*. **58**, 52-61 (1963).
